# COVID-19 in Slovenia, from a Success Story to Disaster: What Lessons Can Be Learned?

**DOI:** 10.3390/life11101045

**Published:** 2021-10-04

**Authors:** Nina Ružić Gorenjec, Nataša Kejžar, Damjan Manevski, Maja Pohar Perme, Bor Vratanar, Rok Blagus

**Affiliations:** 1 Faculty of Medicine, Institute for Biostatistics and Medical Informatics, University of Ljubljana, Vrazov trg 2, 1000 Ljubljana, Slovenia; nina.ruzic.gorenjec@mf.uni-lj.si (N.R.G.); natasa.kejzar@mf.uni-lj.si (N.K.); damjan.manevski@mf.uni-lj.si (D.M.); maja.pohar@mf.uni-lj.si (M.P.P.); bor.vratanar@mf.uni-lj.si (B.V.); 2 Faculty of Sports, University of Ljubljana, Gortanova 22, 1000 Ljubljana, Slovenia; 3 Faculty of Mathematics, Natural Sciences and Information Technologies, University of Primorska, Glagoljaška 8, 6000 Koper, Slovenia

**Keywords:** modeling epidemics, Bayesian inference, discrete renewal process, COVID-19, non-pharmaceutical interventions, reproduction number

## Abstract

During the first wave of the COVID-19 pandemic in spring 2020, Slovenia was among the least affected countries, but the situation became drastically worse during the second wave in autumn 2020 with high numbers of deaths per number of inhabitants, ranking Slovenia among the most affected countries. This was true even though strict non-pharmaceutical interventions (NPIs) to control the progression of the epidemic were being enforced. Using a semi-parametric Bayesian model developed for the purpose of this study, we explore if and how the changes in mobility, their timing and the activation of contact tracing can explain the differences in the epidemic progression of the two waves. To fit the model, we use data on daily numbers of deaths, patients in hospitals, intensive care units, etc., and allow transmission intensity to be affected by contact tracing and mobility (data obtained from Google Mobility Reports). Our results imply that though there is some heterogeneity not explained by mobility levels and contact tracing, implementing interventions at a similar stage as in the first wave would keep the death toll and the health system burden low in the second wave as well. On the other hand, sticking to the same timeline of interventions as observed in the second wave and focusing on enforcing a higher decrease in mobility would not be as beneficial. According to our model, the ‘dance’ strategy, i.e., first allowing the numbers to rise and then implementing strict interventions to make them drop again, has been played at too-late stages of the epidemic. In contrast, a 15–20% reduction of mobility compared to pre-COVID level, if started at the beginning and maintained for the entire duration of the second wave and coupled with contact tracing, could suffice to control the epidemic. A very important factor in this result is the presence of contact tracing; without it, the reduction in mobility needs to be substantially larger. The flexibility of our proposed model allows similar analyses to be conducted for other regions even with slightly different data sources for the progression of the epidemic; the extension to more than two waves is straightforward. The model could help policymakers worldwide to make better decisions in terms of the timing and severity of the adopted NPIs.

## 1. Introduction

The coronavirus SARS-CoV-2 quickly spread from China throughout the world. What initially seemed like someone else’s problem for many people outside China has quickly become everyone’s daily issue. The reason for such rapid global spread of SARS-CoV-2 has been its high basic reproduction number, $R_{0}$ [1], and a large proportion of asymptomatic [2] and mild cases in younger patients [3,4]. The estimates of $R_{0}$ for SARS-CoV-2 vary substantially, depending on the variant of the virus, country and the method of estimation, and stand at about three for the period 2019–2020 [5,6,7,8,9,10,11]. The reproduction number can change in time due to various factors. We denote the (effective) reproduction number of infection by $R_{t}$, where *t* represents time.

In the absence of a vaccine, non-pharmaceutical interventions (NPIs) were one of the few tools available for lowering the reproduction number and slowing down the progression of the pandemic. The general idea behind NPIs is to reduce the number of contacts between individuals or the probability of infection during contact in order to reduce transmission of the virus [10]. The efficiency of NPIs, therefore, depends on the amount by which they decrease the number of contacts and probability of transmission. This depends on the willingness of the general public to follow them or policymakers to enforce them. At the initial stage of the pandemic, NPIs proved successful in Europe, reducing the reproduction number below one [10] and eventually leading to the end of the first wave. During the summer, however, when most NPIs throughout Europe were not in place, the reproduction number increased above one, resulting in a second (autumn) wave of the epidemic. In the period October–December 2020, most countries were implementing NPIs again, but with varying degrees of success.

Among the types of NPIs a special emphasis in our study is put on contact tracing [12]. While the goal of many NPIs is to limit the contacts of the whole population, the idea of contact tracing is to limit the contacts of a targeted subpopulation with a higher probability of infection, as well as to alert contacts to the possibility of infection and offer preventive services [13]. With contact tracing, the goal is to identify and test all potential contacts of each confirmed case, issuing quarantine orders and isolating all subjects with a high probability of infection. This strategy depends strongly on the resources and capacities of the institution performing it as well as ensuring that the quarantine orders are strictly followed. Hence, its implementation, especially during the peak of the epidemic, can be problematic.

In Slovenia, strict NPIs were implemented at a very early stage during the first wave of the epidemic. Public events were banned on 10 March 2020, when there were in total 49 confirmed cases and no deaths or hospitalized patients due to COVID-19. Complete lockdown, including complete closure of schools and child cares, started on 20 March, with 368 confirmed cases (note a rapid increase when compared with 10 March) as well as 46 and 8 patients treated in hospitals and Intensive Care Units (ICUs), respectively, but still with no deaths. Finally, prohibition of movement outside of the municipality of residence was set on 30 March, when there was one death with COVID-19 amongst 802 confirmed cases with 117 and 29 patients being in hospitals and ICUs, respectively. The restrictions started to lift on 30 April, and by 1 June, most restrictions were no longer in place. In total, from the first detected case on 4 March to 30 June, there were on average 88 confirmed cases per 1 million inhabitants per fortnight (MF) and six deaths MF with COVID-19, with the largest number of patients in hospitals and ICUs, being 117 and 36, achieved on 30 March and 10 March, respectively. With a very small attack rate and a small number of deaths, Slovenia was ranked amongst the least affected European countries during the first wave (see, e.g., [14,15]), implying that NPIs were efficient [16,17].

After 30 June, the epidemic started accelerating again. It advanced slowly at first, with on average 145 confirmed cases MF and 2 deaths MF with at most 25 patients in the hospitals and 5 in the ICUs in the period from 1 July to 31 August. This time, the response of the policymakers was slower than during the first wave, adopting the ‘dance’ strategy, i.e., first allowing the numbers to rise and then implementing strict interventions to lower them again [18,19]. In the initial phase of the second wave, the strategy was to rely on active contact tracing and on adopting milder forms of NPIs not in place during the first wave. An example is the use of face masks, which have been obligatory in closed public spaces and outside since 19 September and 16 October, respectively. Since 9 October, complete contact tracing has no longer been possible due to exceeding the capacities. A couple of weeks later, stricter forms of NPIs were being implemented. Prohibition of movement outside of the statistical region of residence was implemented on 20 October. At the same time, curfew (prohibited movement from 9 p.m. to 6 a.m.) was enforced, an example of NPIs not in place during the first wave. Complete lockdown, including complete closure of schools and child care centers, started on 26 October, and prohibition of movement outside of the municipality of residence has been in place since 27 October. However, there have been on average already 1295 confirmed cases MF and 7 deaths MF in the period from 1 July to 25 October with 560 and 88 patients being treated in hospitals and ICUs, respectively, on 25 October. The NPIs seemed to not have slowed down the progression of the epidemic as efficiently as during the first wave, with numbers rising further with on average 3410 cases MF and 60 deaths MF (on several days, the daily number exceeded 55, hence essentially reaching a total death count from the first wave in a mere two days) in the period from 1 July to 1 December with 1299 and 208 patients being treated in hospitals and ICUs, respectively, on 1 December. During December 2020, Slovenia was ranked amongst the countries which have been the most affected by COVID-19 during the second wave [20], although similar, if not stricter, NPIs were being enforced as in the first wave.

Several factors could potentially explain the differences between the two waves. Of these, we focus on contact tracing, timing of the adoption of NPIs and adherence of the public. The following questions are posed:

1. Did contact tracing play an important role in the progression of the second wave in Slovenia?
2. Can the difference in the two waves in Slovenia be explained by the different patterns of the observed general mobility?
3. Can the bad situation in Slovenia in the second wave be attributed to the later adoption of NPIs when compared to the first wave?
4. Are there any alternatives to the ‘dance’ strategy which could have led to slower progression of the epidemic during the second wave?

To answer these questions, we use the modeling approach. Evidence-based modeling approaches have proved successful for answering real-world questions with numerous results which have had important practical consequences [21,22,23,24,25,26,27,28,29]. For example, Ngonghala et al. [29] used a model for the transmission dynamics and control of COVID-19 in a population [30] to show the efficiency of using face masks, a tool now used throughout Europe. We use a semiparametric modeling framework based on a Bayesian approach, which we developed for monitoring the state of the healthcare system and estimation of key parameters of the epidemic [17], adapting it for the purpose of this study. Bayesian inference was introduced as an alternative to the compartmental models [31,32,33] by formulating simple stochastic models which describe the key features of epidemic spread and then using actual data to estimate the parameters of the model [34]. This framework allows reporting statistically sound intervals of uncertainty [17]. In our model, we assume that the transmission intensity of SARS-CoV-2 depends on the number of contacts, using mobility data as a proxy for the number of contacts. The same approach has been commonly used for the COVID-19 pandemic (see, e.g., [35,36,37]). Specifically, we model transmission intensity using data on daily changes of general mobility of the population published in Google mobility reports [38]. Data from this and similar sources (e.g., Apple [39]) were used in other COVID-19 related studies [37,40,41,42,43,44]. Vollmer et al. [37] use the same data source and a similar approach; however, we extend it by using less strict assumptions. We assume, as do Vollmer et al. [37], that changes in mobility can act as a suitable proxy for the changes in behavior induced by the implementation of the NPIs and verify this assumption using empirical data. This enables us to infer the effects of NPIs by looking at mobility instead of the dates of the implementation of NPIs. The effect of NPIs has been extensively studied. Some studies have evaluated the effect of NPIs on mobility using global mobility data [44] or have used questionnaires [45] for assessing the approval of such measures by the general public. Other approaches used parametric [46] or semiparametric [47,48] models for estimating the reproduction number. Some work [49,50] has also relied on external software [51,52] for estimating the effect of NPIs on the reproduction number.

The rest of the paper is organized as follows. In the next section, we thoroughly describe the data sources, the model and different counterfactual scenarios used to illustrate what lessons could be learned from the Slovene example. This is followed by the presentation of the most significant results, discussion and summary of the main conclusions.

## 2. Methodology

The main idea was to adapt the framework proposed by Manevski et al. [17] incorporating many different data sources regarding the state of the epidemic and the healthcare system as well as mobility data from the population. As the different available data sources are at the core of our model, we begin with their description and continue with the detailed presentation of the model. This is followed by a description of the counterfactual analyses. The validation of the model and its computational aspects conclude this section.

### 2.1. Data Sources

The two data sources used were the data about the progression of the Slovene COVID-19 epidemic and the mobility data up to and including 1 December 2020. All data have been split into two waves at 30 June, which will be denoted with subscript m∈{1,2}. This particular day was chosen as it corresponds to the first re-enforcement of the NPIs (see Table S1 showing dates and descriptions of major events important for the progression of the Slovene epidemic).

The first source consists of the following indicators of the state of the epidemic and the healthcare system. We present the notation and add its meaning in parentheses so that the notation is easier to follow. Daily data on the number of confirmed cases is denoted by $P_{t,m}$ (positive cases), number of hospitalized patients by $H_{t,m}$, number of patients in Intensive Care Units (ICU) by $U_{t,m}$, daily number of new patients admitted to hospitals and ICUs by $H_{t,m}^{I}$ (hospital in) and $U_{t,m}^{I}$ (ICU in), respectively, daily number of patients released from hospitals and ICUs by $H_{t,m}^{O}$ (hospital out) and $U_{t,m}^{O}$ (ICU out), respectively, daily number of deaths occurring in hospitals by $D_{t,m}^{H}$ (death from hospital) and daily number of deaths occurring outside of hospitals by $D_{t,m}^{C}$ (death directly from cases), where $t=1,\ldots ,n_{m}$ are days from the start until the end of the wave m∈{1,2}.

Mobility data were obtained from Google Mobility Report [38]. These data show daily relative changes to baseline (the median value during the 5-week period from 3 January to 6 February 2020) of visits and length of stay at different places obtained from users who have opted-in to Location History for their Google Account. Google provides data for six mobility dimensions, which are highly correlated (see S1 File). In order to reduce the problem of collinearity between the mobility dimensions provided by Google and to obtain interpretable dimensions, we include three mobility dimensions in the model, as follows. The first dimension is *workplace* (mobility trend for places of work). This dimension was included since a survey conducted by the National Institute of Public Health suggested that, after household transmissions, the leading origin of transmission in Slovenia is the workplace (17% [53]). The second was *average mobility* (daily average of three dimensions reported by Google: mobility trends for places such as grocery markets, food warehouses, farmers’ markets, specialized food shops, drug stores and pharmacies; mobility trends for public transport hubs such as bus and train stations; and mobility trends for places such as restaurants, cafes, shopping centers, museums, libraries and movie theatres). This was included since it was shown that transmission in day-to-day activities is an important source of infections [35]. We show in the S1 File that using a daily average of the three mobility dimensions when forming the *average mobility* dimension can be justified by formal time series analysis: applying cross-correlation function to the trend of the three dimensions as well as to detrended and deseasonalized data using a standard prewhitening procedure shows that the cross-correlation functions reached very high (positive) values which were the largest at zero lag. The third dimension is *residential* (mobility trend for places of residence). This dimension was included since it is expected to increase when the other dimensions decrease and thus should capture the remaining information not contained in the first two dimensions. Note that the *residential* dimension is not necessarily related to the household transmission which is governed by principles not directly related to mobility. The three mobility dimensions thus obtained are still correlated (see S1 File); therefore, their individual effects could not be disentangled from the model. Prior to including the three mobility dimensions in the model, each dimension was smoothed by taking a 10-day moving average, thus left-aligning the series. The mobility dimensions are denoted by $z_{k,t,m}$, where k=1, 2, 3 represent *workplace*, *average mobility* and *residential* dimensions, respectively, and *t* and *m* are days and waves as above. A 10-day moving average was used since this is the most commonly presumed infectious period for SARS-CoV-2.

**Figure 1:**
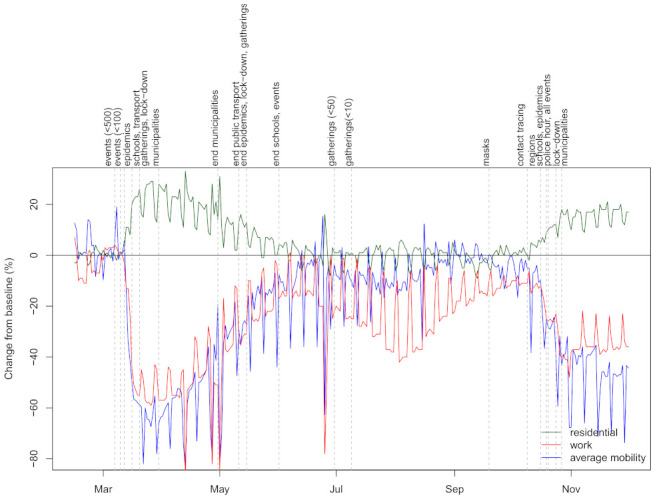
Changes to baseline (%) in mobility dimensions and dates of major government non-pharmaceutical interventions (NPIs).

In Figure 1, we show the three mobility dimensions (not smoothed) together with dates of major NPIs. Large changes of mobility are observed after adoption of NPIs during the first wave, reaching substantially lower (*workplace* and *average mobility*) or higher values (*residential*) as pre-COVID. Even before the last adopted intervention (enforcing prohibition of movements outside the municipality of residence), the changes in mobility started to drop reaching almost pre-COVID values before 1 July. Smaller values for *workplace* and *average mobility* are observed during the summer months due to holidays, as expected. After NPIs were being re-enforced, mobility started to decrease. However, as was similarly observed during the first wave, the mobility stopped decreasing after the final NPI was adopted. Importantly, it can be seen that changes in mobility during the second wave were not as large as during the first wave. There is a clear visual correspondence between the adoption of NPIs and changes in mobility dimensions, which is formalized by performing segmented regression analysis (see S1 File). We show that dates of NPIs correspond to changes in the trend of the three mobility dimensions, thus suggesting that effects of NPIs can be inferred from looking at changes in mobility.

The data about the progression of the epidemic and dates of major government NPIs were obtained using publicly available Slovene COVID-19 data [54]. The population count (also used in the model) was obtained from the Statistical Office of Slovenia [55].

### 2.2. The Model

**Figure 2:**
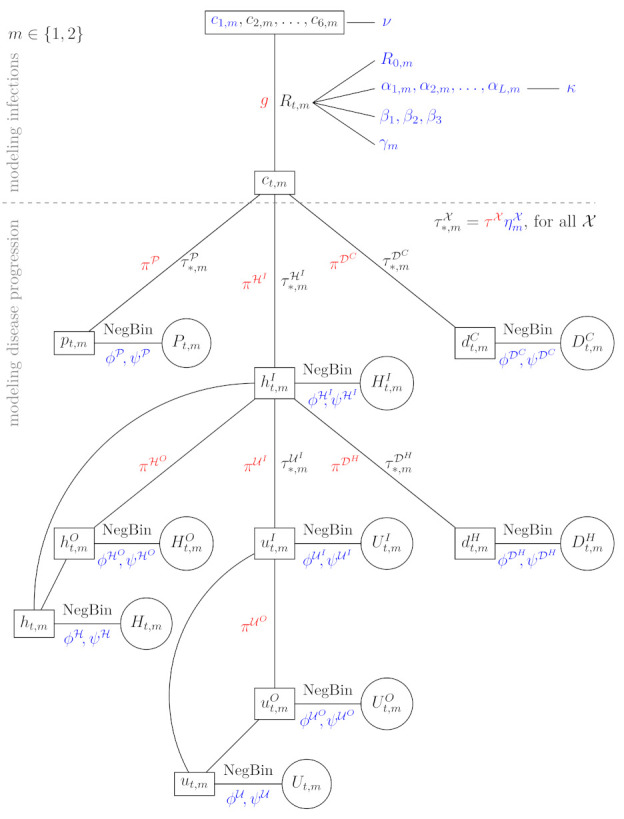
The model for one of the waves m∈{1,2} with data values (in circles); its expected values (in boxes); deterministic parameters in red; and ‘true‘ parameters (for which posterior distributions are estimated) in blue (the parameters with prior distributions) or black (the transformed parameters, including expected values in boxes). See Table 1 for a short description of the quantities and S2 File for more details.

Our proposed model is schematically presented in Figure 2 and is concisely explained in the next subsections with the emphasis on parts that are the key for our goal, i.e., assessing the effect of mobility and contact tracing, whereas all the theoretical details can be found in S2 File. First, we present the part used for modeling infections (above the line border on Figure 2) and then the part for modeling disease progression (below the line border). The first part of the model was crucially modified from [17] to incorporate mobility data, while the second part was adapted to model each wave separately. In Figure 2, we present the model for one of the waves m∈{1,2}, where the data are presented in circles, the deterministic parameters of the model are colored red, while ‘true‘ parameters of the model (for which posterior distributions are estimated) are in blue (the parameters with prior distributions) or black (the transformed parameters, including expected values in boxes). A short description of the quantities used in Figure 2 is given in Table 1.

#### 2.2.1. Modeling Infections

The core of our model is the reproduction number. We allow it to be determined by the mobility data and contact tracing but also account for some potential heterogeneity in the transmission that can not be captured by mobility and contact tracing.

The number of truly infected individuals $c_{t,m}$ (cases) is modeled using a discrete renewal process [10]. We assume that the elapsed time between a person getting infected and subsequently infecting others (i.e., the *generation interval distributiong*) has a fixed distribution in the model. We use gamma distribution as in Flaxman et al. [10] and Manevski et al. [17]; see S2 File for more details and its graphical presentation.

The expected number of truly infected individuals $c_{t,m}$ on a given day *t* during the *m*-th wave is then modeled by

(1) $$c_{t,m}=R_{t,m}\sum_{k=0}^{t-1}c_{k,m}g_{t-k},$$

where $R_{t,m}$ is the time-varying reproduction number modeling the average number of secondary infections over time and $g_{s}$ is the discretization of *g* (see S2 File). Infections at time *t* in wave *m* depend on the number of infections in the preceding days of this wave, first weighted by the discretized generation interval distribution and then scaled by $R_{t,m}$. Note that $c_{t,m}$ has no corresponding data source.

In our model, reproduction number $R_{t,m}$ can be affected by (temporal) herd immunity against SARS-CoV-2, the three mobility dimensions (denoted by $z_{k,t,m}$, k=1,2,3) and by the presence of contact tracing $I_{t,m}$ (where $I_{t,m}$ is a (centered) dummy variable equal to −0.5 if contact tracing was in place on day *t* during the *m*-th wave and 0.5 otherwise). All the other factors that can contribute to the change of the reproduction number are captured in the spline with knots positioned uniformly during the *m*-th wave. This is different from our previous model [17], where knots were positioned at the days of major government changes of policies, as now the mobility and contact tracing directly affect $R_{t,m}$ and the spline should capture the other effects. Moreover, the key is that we are estimating the continuous reproduction number with a continuous estimate via splines (contrary to a piece-wise constant estimate in Flaxman et al. [10]). The reproduction number is then modeled as follows:

(2) $$R_{t,m}=\left(1-\frac{\sum_{k=1}^{t-1}c_{k,m}}{N_{m}}\right)R_{0,m}\left[2f\left(-\sum_{k=1}^{3}\beta_{k}z_{k,t,m}\right)\exp\left(\sum_{l=1}^{L}\alpha_{l,m}s_{k,t,m}\right)\exp\left(\gamma_{m}I_{t,m}\right)\right],$$

where $1-\frac{\sum_{k=1}^{t-1}c_{k,m}}{N_{m}}$ is the term accounting for herd immunity, $N_{m}$ is the size of the population in wave m∈{1,2}, f(x)=1/(1+exp(−x)) is the logistic function (also called the sigmoid function) and $s_{l,t,m}$ is the *l*-th column of the B spline basis matrix for the natural splines with uniformly set knots for the *m*-th wave. We performed sensitivity analysis regarding the number of knots and opted for five knots (see S3 File). Note that the natural splines are linear beyond the final knot, thus enabling predictions in the future. Parameter $R_{0,m}$ is the basic reproduction number with a prior distribution, specified in S2 File. The term accounting for herd immunity was explicitly included in the model to avoid the herd immunity being the main driver of the estimated parameters in (2), when the total number of previously infected is large. We take $N_{1}$ as the population size of Slovenia and define $N_{2}=N_{1}-\sum_{t=1}^{n_{1}}D_{t,1}$, i.e., the population size reduced by the number of deaths during the first wave. Note that $N_{2}$ (which determines how quickly the population determines the herd immunity during the second wave) and $N_{1}$ are almost identical due to very small number of deaths during the first wave; hence, this adjustment is not very important in our application.

Note that we assume the same effects of the mobility in both waves, whereas the effects of contract tracing and other factors via splines may vary between the waves. The logistic function was used as a link between the effect of the combined mobility dimensions and time-varying reproduction number because, due to collinearity, it is not possible to estimate the relative importance of each dimension [37]; for more details, see S2 File. We assume that the effects of interest are independent with some prior distributions, whereas all $\alpha_{l,m}$ have a prior distribution with a common hyperparameter κ. The details on prior specification are in S2 File.

Since the process $c_{t,m}$ cannot start from zero (see (1)), we seed the model for the first six days, separately for each wave, as explained in S2 File (in Figure 2, this is emphasized by showing $c_{1,m},\ldots ,c_{6,m}$ in a separate box).

#### 2.2.2. Modeling Disease Progression

For this part of the model, we used the same techniques as in [17], but we adapted them for modeling two waves where some parameters are wave-specific and others are not. As this part of the model is not crucial for the purpose of our paper (i.e., investigating the effect of mobility and contact tracing on the epidemics) and is a straightforward generalization of our previous model [17], we give only an intuitive summary here and present it thoroughly in the S2 File.

The expected daily number of confirmed cases $p_{t}$, expected daily number of patients admitted to hospitals $h_{t}^{I}$ and the expected daily number of deaths outside of hospitals $d_{t}^{C}$ are modeled directly from cases $c_{t}$. The confirmed cases are intentionally not linked to hospitalized patients or deaths as this data source is the most unreliable one. Once a patient is in the hospital, there are three options: release from the hospital ($h_{t}^{O}$), admission to the ICU ($u_{t}^{I}$) or death ($d_{t}^{H}$). This is incorporated in our model, as presented in Figure 2. There is no link from $u_{t}^{I}$ to death after the admission to the ICU as no data providing this information exists in Slovenia. Furthermore, the expected daily number of hospitalized patients $h_{t}$ is modeled in addition to $h_{t}^{I}$ and $h_{t}^{O}$ due to some inconsistencies in our data sources between the three, and the same principle was followed for ICU. As in [10,17], negative binomial distribution was used when modeling the number of confirmed cases, the number of deaths, etc., allowing that the amount of over-dispersion is determined by the data by specifying the appropriate hyperparameter. All the details on the choice of the distributions, prior distributions and the way we link all the parameters together are given in the Supplementary Material S2 File.

### 2.3. Counterfactual Analyses

By fitting the model to the data, one can learn about the estimated values of the parameters; however, to fully understand their implications and to answer the questions posed in the introduction, we turn to simulations of counterfactual scenarios. To be able to study the effect of the two key variable vectors in our models, i.e., the mobility dimension in time and the contact tracing indicator in time, we simulate hypothetical scenarios in which we change their values and use our model (with the parameters estimated on real data) to study the outcomes.

In all scenarios, we change the actual observed mobility to hypothetical values from a chosen time point *t* onwards. In this way, we simulate a counterfactual scenario in which a certain mobility level was attained at a different point in time than in the observed reality. All scenarios are repeated twice: (a) with contact tracing active as observed in reality (active until 8 October and then discontinued) or (b) kept active throughout the second wave, which can help us in answering Question 1.

**Figure 3:**
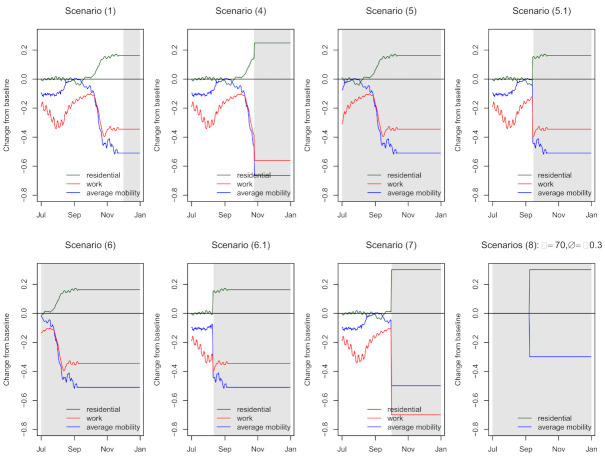
Mobility under different counterfactual scenarios during the progression of the second wave. Scenarios (2) (mobility set to zero for all dimensions) and (3) (*workplace* and *average mobility* dimension set to −1, while *residential* dimension value set to 1) are omitted because of their simplicity. Only one example is shown for scenarios (8) with τ=70 and Δ=−0.3; *work* mobility dimension is not shown since it is exactly the same as the *average mobility* dimension. Shaded areas represent time interval with artificial mobility.

Below, we precisely define the scenarios and explain the reasoning for each choice. We use $z_{2,k,t}^{*}$ to denote the hypothetical value of mobility level *k* (where k= 1 and 2 correspond to *workplace* and *average mobility* dimensions and k= 3 corresponds to *residential* dimension) at time *t* in the second wave. Time is measured in days since the first day of the second wave, which was set to 1 July. The observed data are available until 1 December, i.e., the total number of days in the second wave equals $n_{2}=154$. Based on the model, we also simulate forwards up to 31 December. In all the scenarios, the hypothetical mobility levels $z_{2,k,t}^{*}$ equal the observed levels $z_{2,k,t}$ until a certain time point. Below, we list these time points and the mobility values assumed from then on. All three mobility levels are always changed simultaneously (the levels are correlated and it thus makes no sense to change one, but not the other). Figure 3 illustrates the assumed mobility levels in time for each of the scenarios except scenarios (2) and (3) which are omitted because of their simplicity; shaded areas represent time interval with artificial mobility.

Scenario (1) keeps the same mobility levels throughout the observed period and fixes them to the last observed value for the purpose of the forecast. Scenario (1) (a) thus presents the observed reality, whereas scenario (1) (b) can be used to study the model behavior if the real mobility levels are kept and only the contact tracing is added.

- Scenario (1): $z_{2,k,t}^{*}=z_{2,k,n_{2}}$ for $t>n_{2}$.

Scenarios (2) and (3) represent extreme situations, which in practice could not have occurred, but are included for illustrative reasons. Scenario (2) considers the case where mobility levels are as in ‘normal life’—they remain fixed to the values before the epidemic for the entire duration of the second wave. Scenario (3), on the other hand, assumes no mobility for the whole time period, i.e., the values of *workplace* and *average mobility* dimension are set to the lowest possible value (−1) and the *residential* dimension value is set to 1.

- Scenario (2): $z_{2,k,t}^{*}=0$ for all t≥1;
- Scenario (3): $z_{2,1,t}^{*}=z_{2,2,t}^{*}=-1$; $z_{2,3,t}^{*}=1$ for t≥1.

To answer Question 2 we construct scenario (4), which studies the progression of the second wave if the lockdown was initiated at the same date as in reality (day 118 of the second wave, 26 October) but with a stronger effect on mobility, i.e., reaching the same mobility levels as were reached under the lockdown in the first wave. In terms of *workplace* and *average* mobility dimension, we take the lowest observed value of the first wave, and for *residential* dimension, we take the highest observed value.

- Scenario (4): $z_{2,k,t}^{*}=\min_{1\leq u\leq n_{1}}\left(z_{1,k,u}\right)$ for k=1,2 and $z_{2,3,t}^{*}=\max_{1\leq u\leq n_{1}}\left(z_{1,3,u}\right)$, for t≥118.

Scenarios (5) and (6) consider situations in which the same NPIs were implemented earlier in time as in reality, thus helping us to answer Question 3. It assumes all the mobility levels resulting from NPIs would be equal to those observed but simply moved to an earlier date. In scenario (5), the observed mobility levels are shifted 42 days ahead in time and in scenario (6), the shift is even larger, i.e., 77 days. With this assumption, the lockdown in scenario (5) is implemented on 14 September (day 76 of the second wave), which approximates the stage when lockdown was implemented in the first wave (the day with the closest current number of hospitalized and ICU patients was chosen). Scenario (6) shifts the observed mobility trends to an even earlier stage in the second wave; the date of the lockdown is then August 10 (day 41 of the second wave).

In both scenarios (5) and (6), it is assumed that the course of mobility changes due to NPIs would be exactly the same as observed in reality. After the end of the observed mobility time series, the last value is simply carried forward for the remainder of the interval. Scenarios (5.1) and (6.1) are variants of (5) and (6) in which the NPIs are not gradually working towards lockdown, but instead, it is assumed that the lockdown is implemented without previous notice and the mobility levels change abruptly at the time of lockdown to the levels actually observed after the lockdown date. These variants are thus more similar to other scenarios where mobility levels are changed to hypothetical values not actually observed in the second wave and hence have to be abrupt.

- Scenario (5): $z_{2,k,t}^{*}=z_{2,k,t+42}$ for $1\leq t<n_{2}-42$ and $z_{2,k,t}^{*}=z_{2,k,n_{2}}$ for $t\geq n_{2}-42$;
- Scenario (5.1): $z_{2,k,t}^{*}=z_{2,k,t+42}$ for $76\leq t<n_{2}-42$ and $z_{2,k,t}^{*}=z_{2,k,n_{2}}$ for $t\geq n_{2}-42$;
- Scenario (6): $z_{2,k,t}^{*}=z_{2,k,t+77}$ for $1\leq t<n_{2}-77$ and $z_{2,k,t}^{*}=z_{2,k,n_{2}}$ for $t\geq n_{2}-77$;
- Scenario (6.1): $z_{2,k,t}^{*}=z_{2,k,t+77}$ for $41\leq t<n_{2}-77$ and $z_{2,k,t}^{*}=z_{2,k,n_{2}}$ for $t\geq n_{2}-77$.

The next scenario addresses the situation in which the decision-makers would opt to implement stricter NPIs once it becomes clear that (due to the extent of the virus spread) contact tracing would soon no longer be possible. We set that date to 1 October (day 93 of the second wave) and set the mobility levels to the values that keep the number of positive cases below the capacity limits of the monitoring team (assumed to be around 300 confirmed cases per day).

- Scenario (7): $z_{2,1,t}^{*}=-0.7$, $z_{2,2,t}^{*}=-0.5$ and $z_{2,3,t}^{*}=0.3$ for t≥93.

The above-described scenarios aim to represent the effect of different decisions in time (e.g., what if the same NPIs were implemented a month earlier). Additionally, we study how a certain change in the mobility dimensions’ levels (ignoring the question of how those levels could be reached with NPIs) affect the estimated cumulative number of deaths in the presence or absence of contact tracing. Comparing this to the results of the model where mobility remains at the pre-COVID level ($z_{2,k,t}^{*}=0$ for t≥1) will allow us to answer Question 4 and better understand how the size of mobility change, as well as its timing, affects the results in our model. For this comparison, we run 196 simulation scenarios. First, we pick the time point τ when the mobility levels change from baseline. We consider 14 time points set 10 days apart: τ=10,20,…,140. At the chosen time point τ, the mobility jumps from 0 to Δ, where Δ=−0.1,−0.15,−0.2,−0.3,−0.5,−0.7,−0.9:

- Scenarios (8): $z_{2,k,t}=0$ for all t<τ and all *k* and
  $z_{2,k,t}^{*}=\Delta$ for k=1,2 and $z_{2,3,t}^{*}=-\Delta$ for t≥τ.

We consider the resulting 98 scenarios twice—with contact tracing performed during the entire study period ($I_{2,t}^{*}=-0.5$ for all t≥1) or without any contact tracing ($I_{2,t}^{*}=0.5$, t≥1).

### 2.4. Computational Aspects and Model Validation

The modeling framework has been thoroughly validated in our previous research where sensitivity analysis for the mean parameter of generation interval distribution $\mu^{G}$ was performed [17]. Folded-normal distribution was also compared with other commonly used prior distributions for positive quantities, i.e., gamma and log-normal distributions. Several choices for the over-dispersion parameter of the assumed negative binomial distributions were considered. The differences were not substantial (see [17]). For the model presented in this paper, we additionally performed sensitivity analysis using natural splines with three, four and five degrees of freedom (see S3 File). The results obtained with natural spline with five degrees of freedom were also compared with the results obtained using cubic splines with degrees one, two and three, giving very similar results (these results were obtained using fewer iterations, data not shown). Using natural splines with five degrees of freedom, 5-day and 7-day moving average was also used to smooth the mobility dimensions, obtaining similar results (these results were obtained using fewer iterations, data not shown). All the models fitted the data well with small differences between the observed data and the model’s estimates (see S3 File).

In order to make sure that the effects of mobility, contact tracing, and other effects modeled via the spline term can be identified by the model, we fitted several models excluding some terms from Equation (2). Excluding the mobility, i.e., excluding the term $2f\left(-\sum_{k=1}^{3}\beta_{k}z_{k,t,m}\right)$ from Equation (2), one can observe that the models where mobility is included better fit the data, yielding slightly narrower CIs (see S3 File). We also modeled transmission only as a function of mobility and contact tracing, obtaining worse fit, especially for the first wave, suggesting that the term including the spline can adequately capture the changes in transmission between the two waves not captured by mobility and/or contact tracing (see S3 File). The models with and without mobility were also fitted by omitting contact tracing, i.e., the term $\exp\left(\gamma_{m}I_{t,m}\right)$ in Equation (2). The fit, especially after 9 October, was substantially worse with too-small estimates of the reproduction number (see S3 File) which would result in a too optimistic progression of the epidemic than what is actually observed. This implies that, at least to some extent, the effect of contact tracing, given mobility, can be identified.

Convergence of the models was assessed based on $\hat{R}$ statistics [56], trace plots, estimated posteriors, mean with 50%, and 90% credible intervals (CI) for each chain individually (see S3 File). Credible intervals were obtained using the equal-tailed interval method. Note that the CIs are obtained from the proposed model and not from the estimation of the reproduction number relying only on incidence data and distribution of generation time.

The models were fitted in R (R version 3.6.3 [57]) using R package rstan [58]. The models were fitted using 1600 iterations (800 warmup iterations), 4 chains, setting the target average proposal acceptance probability during standard adaptation period to 0.98 and putting a cap on the depth of the trees that it evaluates during each iteration to 15. The analysis was performed on clusters of CentOS based containers. All the code and data needed to reproduce the results are available on GitHub [59].

## 3. Results

We report a series of selected results for the model based on natural splines with five degrees of freedom; complete results are available as Supplementary Information where results for the other degrees of freedom are also shown (see S3 File and Figure S1). There are large differences in the estimated numbers of infected individuals between the waves with around a 50-fold increase in the average number of newly infected per 1 million inhabitants in 14 days (MF), where this increase is largely due to the later phase of the second wave, i.e., after 26 October (90% CI MF: [207–486], [9616–27,778], [4559–12,901] and [25,498–75,273] MF for the first wave, second wave, early phase of the second wave (1 July to 24 October) and later phase of the second wave (25 October to 1 December), respectively; see S3 File for more results). Note that these large differences are not determined by the reproduction number alone, but it is also crucial how many people are infected at that time (see Figure S1). If a country manages to reduce the reproduction number via NPIs at a time when the number of infected individuals is already high (as in the middle of October during the second wave), the burden on the healthcare system remains substantial. This leads to a presumption that the timing of interventions is of highest importance, which we further investigate with counterfactual analyses.

Counterfactual scenarios (2) and (3) serve as an illustration of what would have happened if people had not changed their behavior at all, or if the mobility had been completely stopped, respectively. The first would have lead to a 5- to 10-fold increase in deaths, while the second would have lead to very few deaths (see Supplementary Information Table S2 and S3 File for more elaborate results).

**Figure 4:**
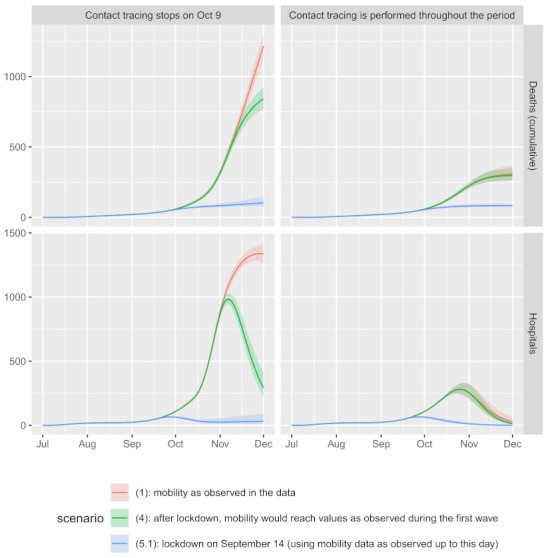
Estimated number of cumulative deaths and the number of patients treated in hospitals during the second wave up to 1 December 2020 under different scenarios (see Section 2.3 for more details regarding the scenarios). Solid lines represent posterior means, shaded areas represent 90% credible intervals (CIs) (see Section 2.4 for more details).

The effect of contact tracing (Question 1) under the observed mobility, scenario (1), is estimated to be large: with contact tracing in place throughout the duration of the second wave, scenario (1) (b), the estimated number of deaths drops from 54 MF (90% CI: [50–57]) under the real situation (i.e., contact tracing is terminated on 9 October; scenario (1) (a)) to 14 MF (CI: [12–16]) and the largest number of patients in the hospitals drops from 1338 (CI: [1256–1430]) to 281 (CI: [245–322]). However, this is only a part of the answer, and to better understand the effect of contact tracing, we turn to Figure 4, where we show the cumulative number of deaths and the number of patients treated in hospitals at each day for the most important selected scenarios: (1)—real, (4)—lockdown mobility as in the first wave and (5.1)—lockdown time as in the first wave. Contact tracing does not have a large effect if the mobility is strongly reduced (see curve (5.1)). On the contrary, if mobility were at high values (scenarios (1) and (4)), then contact tracing would substantially lower the number of deaths and hospitalizations. However, it is debatable if contact tracing is feasible with large mobility due to the fact that, in this case, there will be many individuals infected, thus requiring large capacities to perform contact tracing.

Regarding Question 2, reducing the mobility to the levels observed after the lockdown during the first wave, scenario (4), diminishes the number of deaths and hospitalizations (Figure 4). Specifically, with contact tracing as in reality, scenario (4) (a), the estimated cumulative number of deaths drops to 37 MF (CI: [34–41]) and the largest number of patients in the hospitals drops to 983 (CI: [940–1029]). The earlier implementation of lockdown (Question 3) is even more efficient; see for example scenario (5.1) (a): the cumulative number of deaths with contact tracing as in reality is estimated to 5 MF (CI: [4–7]) and the largest number of patients in the hospitals to 66 [62–70], and observe the non-overlapping CIs even with scenario (4) (a). We observe also that the actual numbers during the first wave are similar to the estimated numbers for scenario (5) (a) (117 in hospital on March 30, estimated 169 (CI: [113–271]); 36 in ICU on March 10, estimated 29 (CI: [19–46]) and 6 deaths MF, estimated 11 (CI: [7–16])). This implies that a similar progression of the epidemic during the first and the second wave could have been observed if NPIs during the second wave had been adopted at a similar stage of the epidemic as they were during the first wave. As expected, implementing the lockdown even earlier (scenarios (6) and (6.1)) further decreases the number of deaths and patients in the hospitals (see Table S2). Implementing the lockdown later could be similarly efficient as observed under scenario (5), but this would require a large decrease in mobility, which in practice would be difficult to achieve; see scenario (7).

**Figure 5:**
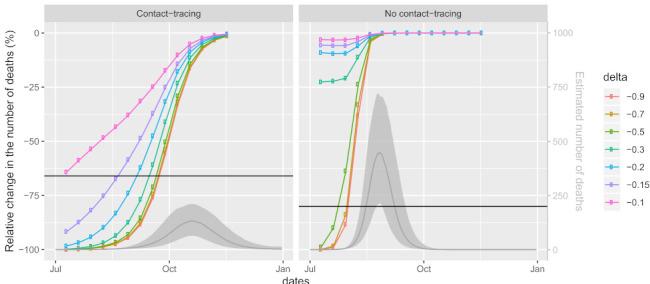
The relative difference (%) of the number of estimated cumulative deaths during the period July 1 to December 31 between two models: the model with the intervention and the model where mobility is at the pre-COVID values throughout the studied period (pre-COVID model). The intervention is adopted at different times - τ=10,20,…,140 (*x*-axis: July 10 corresponding to τ=10, July 20 to τ=20, etc.) assuming various effects on mobility - Δ (different lines, see legend; Δ=−1 would represent a complete stop in mobility). Horizontal black lines show the relative difference between scenario (1) (a), i.e., the scenario based on the observed mobility and contact tracing as well as the pre-COVID model; all points appearing below this line correspond to scenarios where the number of deaths during the study period would be smaller than predicted in the scenario (1) (a). To see at what stage the intervention is applied, grey lines represent posterior means and shaded areas represent 90% CIs for the number of deaths, as estimated by the pre-COVID model (right *y*-axis).

To obtain more insight and alternative scenarios with slower progression of the epidemic (Question 4), we further explore how the number of deaths in the second wave of the Slovene COVID-19 epidemic is associated with three factors—the timing of the intervention, the change of mobility and the implementation of contact tracing—by considering 196 different counterfactual scenarios (8) (see Section 2.3 for more details). The results are summarized in Figure 5, where each point denotes the relative difference (%) of the number of estimated cumulative deaths during the period 1 July to 31 December of a model with the intervention adopted at time τ with its effect on mobility Δ, to the model where mobility is at the pre-COVID values throughout the studied period (pre-COVID model). The *x*-axis shows the day at which the intervention is applied (10 July corresponds to τ=10, 20 July to τ=20, etc.); colors present different changes of mobility (Δ=−1 would represent a complete stop in mobility). To see at what stage the intervention is applied, grey lines and grey shaded areas represent the posterior mean and 90% CI, respectively, for the estimated number of deaths in the pre-COVID model. All models were fitted assuming contact tracing is performed throughout the studied period (left panels) or not at all (right panels). As a benchmark for comparison, we add the actual relative difference observed, i.e., the scenario (1) (a) which is based on the observed mobility levels and contact tracing. The actual gain relative to the estimated number of cumulative deaths was 66% (90% CI [48–70]%) compared to a situation with no changes of mobility but active contact tracing (Figure 5 left panel). When comparing it to the situation without contact tracing (Figure 5 right panel), the gain is even larger, i.e., 80% (CI: [69–82]%). Both values are shown as horizontal lines; all results below these lines correspond to scenarios where the number of deaths is smaller than as predicted in scenario (1) (a).

We can see that effects of the three factors are not independent, as the efficiency of one depends on the values of the others. Furthermore, the effects of timing and change in mobility are both non-linear (see the shape of the curves and the relations between them). In the early and final phases of the epidemic (when the daily numbers of deaths are small), the effect of later implementation of the intervention is smaller than in the middle phase of the epidemic (with high numbers of daily deaths). Thus, during the early phase of the epidemic, delaying the intervention is less important than delaying it by the same amount during the later phase of the epidemic.

The number of averted deaths also depends on the magnitude of the change in mobility, with larger gains observed with larger changes in mobility as expected, but after a certain threshold, a further reduction in mobility does not substantially lower the number of deaths (curves for Δ between −0.5 and −1 are quite similar).

Comparing the situations with and without contact tracing, a clear shift of the curves to the left is observed when contact tracing is not performed. This means that delaying the intervention and/or a lesser reduction of mobility is even more problematic when contact tracing is not performed. Without contact tracing, the curves are steeper, so at a fixed reduction of mobility (e.g., something that would be determined as sustainable), the time of intervention is even more crucial.

The comparison with the model using observed mobility and contact tracing (horizontal lines in Figure 5) reveals that, with contact tracing being performed, a strategy of a mere 10% reduction of mobility throughout the entire study period is as beneficial as the strategy used in reality (where the mobility reduction varied from 0% to almost 50%). Under this scenario, the largest number of positive tests (1409; 90% CI: [243–6261]) would be too large to actually perform contact tracing with the actual capacities of the monitoring team. However, reducing mobility by 15% or 20% throughout the study period would lead to 91% (CI: [82–96]%) and 98% (CI: [96–99]%) reduction in the number of deaths compared to the no-action scenario, respectively. Moreover, these reductions are large enough that contact tracing could be sustained; the largest number of positive tests was estimated to be 475 (CI: [53–3440]) and 53 (CI: [9–232]), respectively. Further reducing mobility would lead to even larger gains, provided that the intervention is not being delayed for too long: if the reduction in mobility had been greater, the delay could be longer. Note, however, that these results depend strongly on the ability to perform contact tracing. Without contact tracing, the reduction in mobility would need to be substantially larger in order to see similar gains (Figure 5 right panel).

## 4. Discussion

In this work, we attempted to understand large differences observed in the progression of the Slovene COVID-19 epidemic between the first and the second wave. Three main reasons have been put forward as possible explanations: the termination of contact tracing, the belated adoption of non-pharmaceutical interventions (NPIs) and the less pronounced reduction of the population’s mobility levels after NPIs compared to the first wave. To study how important these reasons were and how they interacted, we derived a semi-mechanistic Bayesian model linking transmission dynamics of the virus to contact tracing indicators and time-dependent mobility data, informing the model’s parameters using data about the progression of the epidemic (e.g., number of deaths, number of hospitalized patients, etc.). Sequential implementation of NPIs, often within rather short time intervals, combined with the fact that many NPIs are period-specific (may be differently adhered by the general population in one period than in another) withholds the possibility of estimating their individual effects and can potentially lead to noninformative inference. Instead of focusing on dates of NPIs, we therefore turned to study their effect on mobility instead. Our detailed time series analysis suggests that changes in mobility trends can be attributed to the adoption of NPIs; therefore we can infer the effects of NPIs by looking at mobility. A similar conclusion was also reached by Vollmer et al. [37].

Our results imply that all three reasons could have attributed to differences between the waves. Though we have allowed for period-specific effects (parameters α in our model), the simulations using our model imply that implementing a lockdown at a similar stage of epidemic progression as in the first wave would have a similar effect, i.e., keeping the numbers at negligible levels (compared to the actual observed numbers). This would be true even though we take into account that the mobility reduction after lockdown observed in the second wave was not as pronounced as in the first wave. On the contrary, keeping the timing equal to that observed in the second wave, but focusing more on reducing the mobility to a similar level as was observed during the first wave, would not be as effective, as the cumulative death toll was already high by the time of the second wave lockdown. The estimated effect of contact tracing proves to be of high importance in our model. The mobility reduction actually observed in the second wave, coupled with contact tracing, would make a significant difference compared to the actual observed data (without contact tracing). Due to a large number of infected cases at the time of the observed contact tracing termination (9 October), this would however require resources that were beyond the capabilities of the existing monitoring team. If a decrease in the number of infections was attempted, to try and keep the contact tracing active, this should be done early enough. Our simulation shows that such an attempt only a week before the actual termination would require a substantial, possibly unreachable, reduction in mobility, i.e., even higher than the maximum reduction observed during the first wave.

Importantly, the studied effects are not independent of each other. In particular, the curves in Figure 5 are non-linear, clearly indicating that the timing of NPIs is crucial. An equal reduction in mobility has a very different effect on the progression of the epidemic if implemented at an early or at a late stage of the epidemic. This is in line with the results of Loewenthal et al. [36] who concluded, using a simple linear model utilizing mobility data obtained from Apple [39] across all OECD countries, that during the first wave, “delaying initiation of social distancing by one week almost doubles the number of deaths”. Similarly to what is observed in our study, they further conclude that timing of the imposed restrictions is more important than their severity.

Both NPIs and contact tracing reduce the number of contacts; therefore, one could expect that when the frequency of contacts is reduced to a sufficient level, further reduction does not bring much more gain. This is confirmed by our counterfactual scenarios (Figure 4): if the lockdown had been implemented early enough, the reduction of mobility observed in the second wave would suffice, and the addition of contact tracing could not add much to the speed of the decrease in the current number of infected and hospitalized. On the other hand, since the epidemic was at a very high stage at the actual implementation of the lockdown, contact tracing would play a crucial role in turning the curves downwards if it was feasible. This important result is further confirmed in Figure 5: when the epidemic is at an early enough stage, NPIs reducing mobility beyond 50% are unnecessarily strict, and when the epidemic has progressed too far, there is not much left to gain with stricter NPIs. When coupled with contact tracing, the gain of mobility reductions beyond 50% is negligible at all stages of the epidemic.

An important lesson learned in this wave of the epidemic is that the ‘dance strategy’ requires strict timing which is hard to achieve. The idea of this strategy is to allow the number of infected individuals to increase to a certain point and then force their drop with somewhat stricter NPIs [18,19]. This strategy seems to have been the plan for the second wave in most European countries; however, a ‘hammer’ in the form of strong NPIs had to follow in most countries. As our results show, the time windows for action are rather narrow and hence very hard to target, as the information on the number of actually infected individuals is only obtained indirectly and with an important time lag. In addition, achieving a certain reduction in mobility may not be so easy in practice and needs some time to implement. There may be several reasons why Slovenia turned out to be one of the first proofs that the strategy did not work, but a simple fact is that its small population implies a large variability of daily data. Therefore, an even longer period is required to discern actual trends from random variability. Adding to this, the general public opinion in Slovenia being ‘rather sorry than safe’, the required timing becomes impossible to stick to. A belated action on the other hand requires much stricter NPIs and at the same time poses an unavoidable extra death toll and burden to the healthcare system.

As an alternative worth considering, we illustrate the effect of scenarios with less strict mobility reduction throughout the epidemic (Figure 5). We show that even a mere 15% reduction of mobility would, if started at the beginning and maintained for the entire duration of the second wave and coupled with contact tracing, imply a 91% (90% CI [82–96]%) relative change in the number of deaths until 31 December compared to the no-action scenario. This is much less than the 66% (90% CI [48–70]%) that we managed to gain in the same period with the actual observed data, despite the fact that mobility reduction varied from 0 to almost 50%. There are two important points that enable these results. First, the timing is crucial as even minor delays require much more extreme reductions in mobility, and longer delays cannot be made up for. The second crucial assumption is that contact tracing is kept active throughout the period, and without it, the reductions in mobility need to be substantially greater. This assumption seems to be entirely realistic with the 20% mobility reduction strategy, as the expected number of positive tests reaches its maximum at 53 per day (90% CI: [9–232]). The 15% mobility reduction strategy may be feasible as well, as the maximum expected number of positive tests per day still remains at the upper limit of the capacities of the monitoring team (475, 90% CI: [53–3440]). In any case, the question of whether such a strategy is sustainable in practice and sensible from other points of view (e.g., economics) as well as whether such a strategy could be further optimized is beyond the scope of our study.

Our findings of a potentially large efficacy of contact tracing seem to contradict the conclusion of Ngonghala et al. [29] who conclude that: “quarantine and contact tracing have marginal impact in minimizing COVID-19 burden”. However, looking closer at their results, we find: “since self-isolation and isolation in hospitals have been implemented at high effectiveness levels in both the state of New York and in the entire US, the mass quarantine of suspected cases may not be a cost-effective public health strategy for combating the spread of COVID-19 in both New York state and the entire US”. In other words, self-isolation, i.e., low mobility level of a targeted subgroup of individuals who are infected or highly likely to be infected is crucial, but contact tracing attempts at a very high stage of epidemic progression may be futile, which is completely in line with our observations.

The study has several limitations. First, as with any model, several assumptions are made. Our model is constructed in a way that as little information as possible is pre-assumed, so our results are mainly driven by the data and not so by the assumptions. This has been further ensured by comprehensive sensitivity analyses that ensure that the impact of the change in the assumptions on the final results is negligible.

Further assumptions stem from the availability and quality of the data. Given the lack of any other reliable source, we resorted to Google mobility data as a proxy for the actual mobility. One needs to be aware that this data may not be representative of the entire Slovene population since it only covers individuals who have opted-in to Location History for their Google Account. Nevertheless, the fact that other studies using similar data reached similar conclusions [37,40,41,42,43] gives us confidence into our results. Due to large collinearity between mobility dimensions provided by Google, the non-identifiability has forced us to merge several reported dimensions (mobility trends for grocery markets, food warehouses, pharmacies, etc.; mobility trends for public transport hubs; and mobility trends for restaurants, cafes, etc.) into a single dimension which we name *average mobility*. The merging is performed by simply calculating the average. A time series analysis has been conducted to justify the use of this simple approach and ensure that there has been no substantial loss of information due to such merging. Additionally to *average mobility* in the model, we also include *workplace* (mobility trend for places of work) and *residential* (mobility trend for places of residence) dimensions. The three included mobility dimensions are associated (as shown by our time series analysis), preventing us from estimating their individual effects.

Several NPIs and other factors which might be related to transmission intensity but are not directly affecting mobility (or contact tracing), e.g., wearing face masks, could also potentially explain the observed differences between the two waves. All these effects are not modeled directly but rather captured in the model by allowing the reproduction number to change via the spline term independently of mobility and contact tracing indicator.

Age is known to be an important risk factor for death and severity of the disease in COVID-19 patients [60], and care had to be taken to avoid the potential bias resulting in different age distributions in the two waves. Unfortunately, complete and reliable age-stratified data were not available. To account for these potential differences, the model thus allows that the transition probabilities between states are different in the two waves. Importantly, their estimation is data-driven by specifying the respective prior distributions. Another feature of our model which helps reducing this bias is via the additional term that captures the variability which is not explained by mobility and contact tracing. This may particularly help with the bias due to changes in the age distribution within the wave which might cause over- or under-estimation of some quantities. Nevertheless, without this data, it is impossible to judge how efficient this bias reduction is, so the lack of age-specific data remains an important limitation of the model. If complete and reliable age-stratified data were available, the proposed model could straightforwardly be adapted by using similar ideas as presented in Manevski et al. [17].

An important limitation of the study is also that this is an observational study with all the NPIs and all the data available on the level of the whole country only. While there have been attempts during the second wave to implement NPIs at different time points in different regions, they were short-lived in practice, as the speed of progression of the epidemic at that time quickly made the situation in all regions critical, forcing maximal interventions throughout the country. While some information can be gained from the fact that we have observed two waves and some confidence in the results can be drawn from the good fit and the seemingly unimportant residual heterogeneity, the interpretation of all the results must be careful. As an example, consider the effect of contact tracing. This was terminated at a crucial phase of the epidemic and is hence estimated to have an important effect. However, there might be an interaction between the effect of contact tracing and the mobility levels, and the effect may be changing in time, but this is not estimable from the given data. In addition, if NPIs were implemented at different stages of the epidemic, this could have induced any sequence of other events that could not be taken into account in our model. Therefore, all the counterfactual analyses in our study should only be understood as an important step towards understanding the past events, the model properties and the possible consequences of the decisions, and no causal conclusions should be attempted based on this work.

Another potential concern is that the difference between the two waves is not driven by human behavior or interventions but merely by the fact that during the second wave, we are dealing with more infected individuals. Here, there are three potential points of concern. The first point is that at the end of the first wave, there can be a non-negligible number of infected which are carried over to the second wave, where looking only at the effective reproduction number would not be meaningful. For our case, we believe that this was not a major concern since the number of infected at the end of the first wave was negligible. Another evidence for this was our counterfactual scenario (5), where lockdown was simulated at a similar stage of epidemic progression as in the first wave, where similar progression of the two waves was observed. The second point is about the potential collapse of the healthcare system due to a large number of infected individuals leading to worse progression of the epidemics. In our case, this was not an issue since the healthcare system quickly adapted to the situation, giving extra resources to treating COVID-19 patients and greatly extending the potential capacities which were not overreached even during the peak of the second wave. The final point is that the difference between the two waves could also be attributed to differences in testing strategies, thus detecting more cases during the second wave. For this reason, we set up the model in such a way that, as explained previously, the transition probabilities are data-driven and allowed to change, and also importantly, the number of confirmed cases is given less weight when estimating the parameters (recall that this data source is completely separated from the other sources).

Performing a study using data for a single country prevents us from inferring that the same effects would be or could be observed elsewhere. However, the modeling framework developed to analyze Slovene COVID-19 data could be applied to other countries and/or regions (even if the data sources would be somewhat different from ours) to further investigate and enable policymakers to make better data-driven decisions in the future. For this purpose, we offer the code, where only basic R programming skills are required. However, as with any complex Bayesian model, the fitting procedure takes quite some computational resources. For example, the analysis took 10 days on a cluster of CentOS-based containers. The large computational burden was, however, mainly due to considering more than 200 counterfactual scenarios and less so due to fitting the models. The questions posed in the paper could also be answered using compartmental models and their modifications incorporating mobility and NPIs, which would require some additional assumptions. It would be interesting to compare the results of the two approaches.

## 5. Conclusions

The mobility data have proven to be a useful proxy for the effect of the NPIs on the contacts between individuals that enable transmission of the virus and can thus help us understand the progression of the epidemic.

The key reason for the high death toll and health burden of the epidemic in Slovenia seems to be the belated response in terms of activating a strong enough reduction in mobility. The lost time cannot be made up for regardless of the strictness of NPIs, and while achieving the same mobility reduction in the second wave as was observed in the first wave would significantly change the course of the epidemic, the cumulative number of deaths would still be high. A particularly important point seems to be the ability to sustain contact tracing, which is another factor that demands strict timing of the interventions.

As an alternative to attempting to target the right timing and jumping between extremes, a strategy of fixing the mobility for the entire period of the epidemic was considered. The results show that a permanent 15–20% decrease in mobility coupled with contact tracing suffices to keep the epidemic under control. This corresponds to the levels actually achieved in the summer and may hence present a realistic alternative.

The model developed for the purpose of this study could be used to conduct similar analyses across other countries (even if data sources are somewhat different from ours), whereby the analysis is not limited to two waves only. If complete and reliable age-stratified data were available, the modeling framework could be adapted to consider this important information which was missing in the model presented herein. The results of such analyses could help policymakers make better data-driven decisions in terms of timing and severity of NPIs adopted to control the spread of the epidemic.

## Figures and Tables

**Table 1:** A short description of the quantities presented in Figure 2. For more details, see S2 File.

Notation	Description
*m*	wave (first, second) of the epidemic
*t*	day in the wave
$R_{0,m}$	basic reproduction number
$R_{t,m}$	(effective) reproduction number at specific day *t* in wave *m*
$\alpha_{l,m}$, l=1,…,L	parameters relating (unknown) factors other than mobility and contact tracing to $R_{t,m}$ (see Equation (2))
κ	hyperparameter tying together $\alpha_{l,m}$
$\beta_{k}$, k=1,2,3	parameters relating mobility to $R_{t,m}$ (see Equation (2))
$\gamma_{m}$	parameter for the contact tracing indicator (see Equation (2))
$c_{t,m}$	expected number of truly infected individuals
$p_{t,m}$	expected number of positive cases
$h_{t,m}^{I}$	expected number of admissions to hospitals
$h_{t,m}^{O}$	expected number of releases from hospitals
$h_{t,m}$	expected number of hospitalized patients
$u_{t,m}^{I}$	expected number of admissions to ICUs
$u_{t,m}^{O}$	expected number of releases from ICUs
$u_{t,m}$	expected number of patients treated in ICUs
$d_{t,m}^{H}$	expected number of deaths in hospitals
$d_{t,m}^{C}$	expected number of deaths outside the hospitals
NegBin	negative binomial distribution
$P_{t,m}$	number of positive cases, modeled via negative binomial distribution with mean $p_{t,m}$, similarly for other quantities
$\phi^{X}$	parameter which determines the amount of over-dispersion (i.e., the additional variance of the negative binomial distribution above that of the Poisson distribution)
$\psi^{X}$	hyperparameter behind $\phi^{X}$
$\pi^{X}$	elapsed time between two successive states (e.g., $\pi^{P}$—time from infection to a positive test)
*g*	elapsed time between a person becoming infected and subsequently infecting others
$\tau_{*,m}^{X}$	probability of transition to a successive state (e.g., $\tau_{*,m}^{P}$—ratio of cases that are being tested)
$\tau^{X}$	fixed, represents a guess for the probability of transition to a successive state, chosen in exploratory fashion as reported in S2 File
$\eta_{m}^{X}$	noise around $\tau^{X}$ which allows that $\tau_{*,m}^{X}=\tau^{X}\eta_{m}^{X}$ is determined by the data (note that $\eta_{m}^{X}$ has a prior distribution)
ν	hyperparameter tying together $c_{1,m},\ldots ,c_{6,m}$ which are used for seeding the model

## Data Availability

Publicly available, aggregated, de-identified data were analyzed in this study. All data and the code needed to reproduce the results of this study are available on GitHub: https://github.com/damjanm/COVID-19-Slovenia-mobility.

## Supplementary Materials

The following are available online at https://www.mdpi.com/article/10.3390/life11101045/s1, S1 File: Association between mobility dimensions and non-pharmaceutical interventions. Results of time series analysis of the mobility dimensions and their association with non-pharmaceutical interventions (html); Table S1: Non-pharmaceutical interventions in Slovenia during first and second wave of COVID-19 epidemic. The table with non-pharmaceutical interventions in Slovenia during first and second wave of COVID-19 epidemic was obtained and translated from “Covid-19 Tracker Slovenia” open-source all-Slovene project website [54]. Each NPI is supported by a web address from Slovene government (html); S2 File: Details about the model’s parameters and assumed distributions. Explicit explanation of the part of the model for disease progression: table for the chosen values for the deterministic parameters and figure showing the assumed distributions of various times used in our analyses (pdf); S3 File: Complete results. Complete results of all fitted models including also sensitivity analyses and information about convergence of the models (html); Figure S1: Difference between the two waves as predicted by the model. The figure shows the estimated daily number of infections (left), time-varying reproduction number (middle) and daily number of hospitalized patients (right) during the first wave (first row) and second wave up to 1 December 2020 (second row), where shaded areas represent the estimated 50% and 90% credible intervals. Note a difference in the scales of the *y*-axes for the first and the second wave (pdf); Table S2: Summary for counterfactual models (1)–(7). The table shows the estimated number of cumulative deaths per 1 million inhabitants per 14 days (MF) and the largest number of patients treated in hospitals and ICUs during the second wave up to 1 December 2020 under counterfactual scenarios (1)–(7) (pdf).
