# Supplementary material for: COVID-19 in Slovenia, from a Success Story to Disaster: What Lessons Can Be Learned?
Source: Life (Basel). 2021 Oct 4;11(10):1045. doi: 10.3390/life11101045 (PMC8541564; doi:10.3390/life11101045)
Supplement: Supplementary file 1 [file life-11-01045-s001.zip › S1_file.html]

COVID-19 in Slovenia, from a success story to disaster: what lessons can be learned? Supplementary material: Time-series analysis


# COVID-19 in Slovenia, from a success story to disaster: what lessons can be learned? Supplementary material: Time-series analysis

#### Nina Ružić Gorenjec, Nataša Kejžar, Damjan Manevski, Maja Pohar Perme, Bor Vratanar, Rok Blagus

#### July-2020

# 

## Google mobility data: time series analysis

Here, we show the results for the time series analysis of Google mobility data. For each mobility dimension, we estimated the trend using the moving averages method. We decided to evaluate overlapping trends between different categories with a cross-correlation function. The results show that the cross-correlation functions reach the highest absolute value at around zero lag (Figure 1). Four categories are positively correlated at lag zero: retail & recreation, grocery & pharmacy, transit stations, and workplaces. We also analyzed mobility data for each category using the SARIMA model (Box & Jenkins, 1970). Based on the graphical inspection of (partial) autocorrelation functions we decided to fit the same SARIMA \((0,1,4)(0,1,1)\_7\) model to all movement categories. All models fitted the data adequately (see Figure 1). Lastly, we calculated the cross-correlation function on detrended and deseasonalized data using a standard prewhitening procedure (Bisgaard & Kulahci, 2011). The correlations between time series are small and negligible at non-zero lags. In line with previous results, the cross-correlation functions reach the highest absolute value at zero lag (Figure 1). The zero-lag correlation is positive between the same four categories as before. The results suggest that these four time series share a similar data-generation model. We could, therefore, at each time point average four categories to achieve more parsimonious results without losing much information. In practice, we decided to join only the following categories: retail & recreation, grocery & pharmacy, and transit stations.

**Figure 1.** The cross-correlation functions for five mobility dimensions. The cross-correlation functions for trend data are shown above the main diagonal. The cross-correlations functions for prewhitened time series are shown below the main diagonal. The autocorrelation functions of residuals are shown on the main diagonal. Red lines represent correlations at zero lag.

### References

Bisgaard, S., & Kulahci, M. (2011). *Time series analysis and forecasting by example*. John Wiley & Sons.

Box, G., & Jenkins, G. (1970). *Time Series Analysis: Forecasting and Control*. San Francisco: Holden-Day.

## Association between mobility and NPIs

Here we show the results of interrupted time series regression (Bernal, Cummins, and Gasparrini 2017). First, we test, for each NPI, if the trend in mobility changes after adopting the NPI. For this purpose we assume a continuous trend (i.e joinpoint regression) and set breakpoints to dates of NPIs. The results are shown in Table below, from where we can conclude that NPIs are associated with changes in mobility dimensions.

Results of joinpoint regression with poistion of breakpoints determined by NPIs

|  | work | residential | average mobility |
| --- | --- | --- | --- |
| events (<500) | -9.24 (1.17) \*\*\* | 3.34 (0.32) \*\*\* | -9.08 (0.74) \*\*\* |
| events (<100) | 5.13 (2.92) | -2.09 (0.8) \*\* | 3.33 (1.86) |
| epidemics | -0.65 (2.76) | 0.35 (0.76) | 1.57 (1.76) |
| schools, transport | 4.68 (1.62) \*\* | -1.55 (0.45) \*\*\* | 3.97 (1.03) \*\*\* |
| gatherings, lock-down | -0.32 (0.95) | -0.27 (0.26) | 0.67 (0.61) |
| municipalities | 0.6 (0.27) \* | -0.25 (0.07) \*\*\* | 0.65 (0.17) \*\*\* |
| end municipalities | 1.52 (0.24) \*\*\* | -0.16 (0.07) \* | 0.94 (0.15) \*\*\* |
| end public transport | -1.97 (0.74) \*\* | -0.05 (0.2) | -0.86 (0.47) |
| end epidemics, lock-down, gatherings | 0.65 (0.67) | 0.25 (0.18) | -0.26 (0.43) |
| end schools, events | -0.96 (0.16) \*\*\* | 0.18 (0.05) \*\*\* | -0.48 (0.1) \*\*\* |
| gatherings (<50) | -0.65 (0.21) \*\* | 0.27 (0.06) \*\*\* | -0.24 (0.13) |
| gatherings(<10) | 1.21 (0.17) \*\*\* | -0.2 (0.05) \*\*\* | 0.35 (0.11) \*\* |
| masks | -0.06 (0.09) | 0.29 (0.03) \*\*\* | -0.78 (0.06) \*\*\* |
| regions | -0.19 (1.39) | 0.16 (0.38) | 0.47 (0.89) |
| schools, epidemics | 0.23 (1.7) | -0.55 (0.47) | -0.32 (1.08) |
| lock-down | 2.66 (1.54) | -0.12 (0.42) | 2.98 (0.98) \*\* |
| municipalities | -0.77 (0.93) | -0.24 (0.26) | -1.04 (0.59) |
| contact tracing | -2.02 (0.43) \*\*\* | 0.54 (0.12) \*\*\* | -1.73 (0.27) \*\*\* |

Next, we use the procedure proposed by Bai and Perron (2003) to estimate the optimal number of breakpoints in segmented regression using Bayesian Information Criterion (BIC) to determine the optimal number of breakpoints and their position; note that information about the dates of NPIs is not used in this analysis. The results are shown in Figure below. Using BIC, 4 segments were found for *average mobility* and *workplace* dimensions. The 95% confidence interval (CI) for the position of the first breakpoint corresponds to school closure in both examples, observing substantially lower values afterwards. The next determined changepoint corresponds to the end of the NPIs when looking at the *average mobility*, while for *workplace* this is observed later, during the time of when (mild) NPIs started to be adopted again. The final changepoint again agrees for *average mobility* and *workplace* with 95% CI for the breakpoint corresponding to lock-down, but covering also other NPIs. For *residential* dimension 6 breakpoints were identified, with the positions of the changepoints again corresponding to dates of adopted (or lifted) NPIs.

**Figure 2.** Results of computation of breakpoints for mobility dimensions

### References

Bai, Jushan, and Pierre Perron. 2003. “Computation and Analysis of Multiple Structural Change Models.” Journal of Applied Econometrics 18 (1): 1–22.

Bernal, James Lopez, Steven Cummins, and Antonio Gasparrini. 2017. “Interrupted Time Series Regression for the Evaluation of Public Health Interventions: A Tutorial.” Int J Epidemiol. 46 (1): 348–55.
