# Supplementary material for: COVID-19 in Slovenia, from a Success Story to Disaster: What Lessons Can Be Learned?
Source: Life (Basel). 2021 Oct 4;11(10):1045. doi: 10.3390/life11101045 (PMC8541564; doi:10.3390/life11101045)
Supplement: Supplementary file 1 [file life-11-01045-s001.zip › S2_file.pdf]

COVID-19 in Slovenia, from a success story to disaster: what lessons can be learned?

Supplementary Information: Details about the Model

Nina Ružić Gorenjec, Nataša Kejžar, Damjan Manevski, Maja Pohar Perme, Bor Vratanar, Rok Blagus

S1 Notation

Let $\mathcal{N}(\mu, \sigma)$ denote a normal distribution with mean μ and standard deviation σ. We say that X follows a folded-normal distribution, denoted by $\mathcal{N}^+(\mu, \sigma)$, if $X = |Y|$ and $Y \sim \mathcal{N}(\mu, \sigma)$. Let $\mathrm{Unif}(a, b)$ denote a continuous uniform distribution on $[a, b]$, let $\Gamma(\mu, \xi)$ denote a gamma distribution with mean μ and coefficient of variation ξ, and let $\mathrm{Exp}(\lambda)$ be the exponential distribution with mean $1/\lambda$. Using $\mathrm{NegBin}(\mu, \mu + \frac{\mu^2}{\phi})$ we denote the negative binomial distribution with mean μ and variance $\mu + \frac{\mu^2}{\phi}$. If h is the density of a positive random variable, then $\{h_s\}_{s=1}^{\infty}$ denotes its discretization via

$$h_1 = \int_0^{1.5} h(t)dt, \quad h_s = \int_{s-0.5}^{s+0.5} h(t)dt \text{ for } s = 2, 3, \ldots \tag{1}$$

S2 Modeling infections

In this section, we fill in the details for the part of the model used for modeling infections which is presented in the paper.

Recall that

$$c_{t,m} = R_{t,m} \sum_{k=0}^{t-1} c_{k,m} g_{t-k}. \tag{2}$$

Generation interval distribution g is distributed as $g \sim \Gamma(\mu^{\mathcal{G}}, \xi^{\mathcal{G}})$. We use as in Flaxman et al. [1] and Manevski et al. [2] $\mu^{\mathcal{G}} = 6.5$ and $\xi^{\mathcal{G}} = 0.62$ (see also Figure S1 for graphical presentation). Furthermore, g_s is the discretization of g via (1).

Figure S1: Assumed generation interval distribution (g - upper left) and assumed distributions of various times $\pi^{\mathcal{X}}$ (see Section S3) used in the model; note different scales.

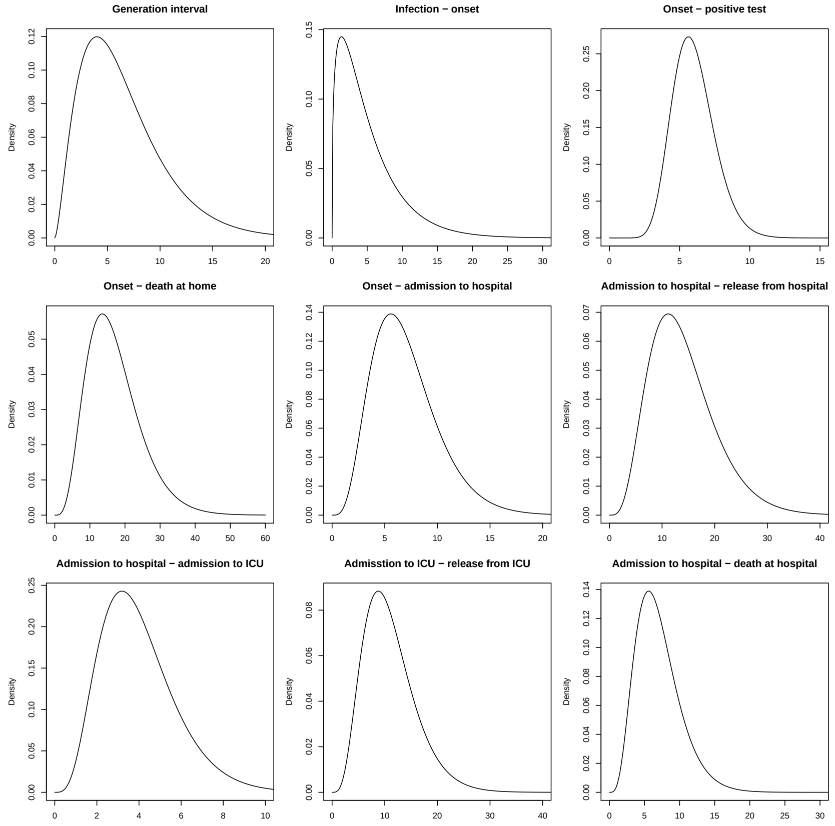

Recall that we model the reproduction number as

$$R_{t,m} = \left(1 - \frac{\sum_{k=1}^{t-1} c_{k,m}}{N_m}\right) R_{0,m} \left[2f\left(-\sum_{k=1}^{3} \beta_k z_{k,t,m}\right) \exp\left(\sum_{l=1}^{L} \alpha_{l,m} s_{k,t,m}\right) \exp\left(\gamma_m I_{t,m}\right)\right]. \tag{3}$$

The basic reproduction number $R_{0,m}$ has a prior distribution (as in [1] and [2])

$$R_{0,m} \sim \mathcal{N}^+(3.28, 0.25). \tag{4}$$

Note that the term $2f$ in (3) ensures that the multiplicative effect of the mobility on the basic reproduction number $R_{0,m}$ is between 0 and 2, where 1 represents no effect. We assume that the effects of interest when modelling $R_{t,m}$ are independent with prior distributions

$$\begin{aligned} \beta_k &\sim \mathcal{N}(0, 0.5), \quad k = 1, 2, 3, \\ \gamma_m &\sim \mathcal{N}(0, 0.5), \quad m = 1, 2. \end{aligned} \tag{5}$$

On the other hand, all $\alpha_{l,m}$ have a prior distribution with a common hyper-parameter κ:

$$\begin{aligned} \alpha_{l,m} &\sim \mathcal{N}(0, \kappa), \quad l = 1, \dots, L,\ m = 1, 2, \\ \kappa &\sim \mathcal{N}^+(0, 0.5). \end{aligned} \tag{6}$$

As in [1] and [2], we assume that seeding of new infections begins 30 days before observing 10 cumulative deaths. From this date, we seed the models with six sequential days $c_{1,m}, \dots, c_{6,m}$ where we chose

$$c_{1,m} = \dots = c_{6,m} \sim \text{Exp}(1/\nu), \quad \nu \sim \text{Exp}(0.03). \tag{7}$$

S3 Modeling disease progression

Here we thoroughly present the part of the model for disease progression, which was only intuitively summarised in the paper. We used the same techniques as in [2], but we adapted them for modeling two waves where some parameters are wave specific and others are not.

The number of confirmed cases is modeled via

$$P_{t,m} \sim \text{NegBin}\left(p_{t,m}, p_{t,m} + \frac{p_{t,m}^2}{\phi^{\mathcal{P}}}\right), \tag{8}$$

where p_t is the expected number of confirmed cases that is modeled mechanistically from cases c_t as

$$p_{t,m} = \tau^{\mathcal{P}}_{*,m} \sum_{k=1}^{t-1} c_{k,m} \pi^{\mathcal{P}}_{t-k}. \tag{9}$$

Parameter $\phi^{\mathcal{P}}$ is common throughout all times and both waves, it determines the amount of over-dispersion (i.e. the additional variance of the negative binomial distribution above that of the Poisson distribution), and has prior distributions

$$\phi^{\mathcal{P}} \sim \mathcal{N}^{+}(0, \psi^{\mathcal{P}}), \quad \psi^{\mathcal{P}} \sim \mathcal{N}^{+}(8, 2). \tag{10}$$

The ratio of cases that are being tested is represented by $\tau^{\mathcal{P}}_{*,m}$, so it varies between the waves. We model it as

$$\tau^{\mathcal{P}}_{*,m} = \tau^{\mathcal{P}} \eta^{\mathcal{P}}_{m}, \tag{11}$$

where $\tau^{\mathcal{P}}$ is fixed (represents a guess for the ratio of cases being tested, chosen in exploratory fashion as reported in Table S2) and $\eta^{\mathcal{P}}_{m}$ is noise around it (the data inform this part which is allowed to change between the waves) with prior distribution

$$\eta^{\mathcal{P}}_{m} \sim \mathcal{N}^{+}(1, 0.5). \tag{12}$$

Finally, $\pi^{\mathcal{P}}_{s}$ is discretized distribution for time from infection to a positive test $\pi^{\mathcal{P}}$. It is deterministic and the same for both waves as it is disease specific. We assume that it is a sum of two independent times: infection to onset $\Gamma(\mu^{\mathcal{O}}, \xi^{\mathcal{O}})$ and onset to positive test $\Gamma(\mu^{\mathcal{P}}, \xi^{\mathcal{P}})$, where the parameters of gamma distributions are deterministic and chosen as reported in Table S2 (parameters for infection to onset are set as in [1, 2], while others depend on testing strategies and were therefore chosen in exploratory fashion).

To sum up, the number of confirmed cases during the m-th wave $p_{t,m}$ is the sum of the past infections $c_{k,m}$ weighted by their probability of transition from $c_{k,m}$ to $p_{t,m}$.

The rest of the data are incorporated in the model following the same logic as $P_{t,m}$, where we assume the conditional independence of different data sources and waves given the model parameters (for more details see Section S4). The formulas for expected number of individuals at a specific state can be unified through

$$\tau^{\mathcal{X}}_{*,m} \sum_{k=1}^{t-1} x_{k,m} \pi^{\mathcal{X}}_{t-k}, \tag{13}$$

where $x_{k,m}$ is an appropriate expected number of individuals at a previous state (as in the schematic presentation of the model in the paper), $\tau^{\mathcal{X}}_{*,m}$ represents the ratio of people moving from one state to another, and $\pi^{\mathcal{X}}$ is the

Table S1: Modeling the expected number of individuals at a specific state (first column) from the expected number of individuals at a previous state $x_{k,m}$ (third column, see also figure of the model from the paper) via formula (13).

quantity	notation	$x_{k,m}$	$\pi^{\mathcal{X}}$	$\tau^{\mathcal{X}}_{*,m}$
positive cases	$p_{k,m}$	$c_{k,m}$	$\Gamma(\mu^{\mathcal{O}}, \xi^{\mathcal{O}}) + \Gamma(\mu^{\mathcal{P}}, \xi^{\mathcal{P}})$	$\tau^{\mathcal{P}}\eta^{\mathcal{P}}_m$
hospital in	$h^I_{k,m}$	$c_{k,m}$	$\Gamma(\mu^{\mathcal{O}}, \xi^{\mathcal{O}}) + \Gamma(\mu^{\mathcal{H}^I}, \xi^{\mathcal{H}^I})$	$\tau^{\mathcal{H}^{\mathcal{I}}}\eta^{\mathcal{H}^{\mathcal{I}}}_m$
hospital out	$h^O_{k,m}$	$h^I_{k,m}$	$\Gamma(\mu^{\mathcal{H}^O}, \xi^{\mathcal{H}^O})$	1
ICU in	$u^I_{k,m}$	$h^I_{k,m}$	$\Gamma(\mu^{\mathcal{U}^I}, \xi^{\mathcal{U}^I})$	$\tau^{\mathcal{U}^{\mathcal{I}}}\eta^{\mathcal{U}^{\mathcal{I}}}_m$
ICU out	$u^O_{k,m}$	$u^I_{k,m}$	$\Gamma(\mu^{\mathcal{U}^O}, \xi^{\mathcal{U}^O})$	1
death in hospitals	$d^H_{k,m}$	$h^I_{k,m}$	$\Gamma(\mu^{\mathcal{D}^H}, \xi^{\mathcal{D}^H})$	$\tau^{\mathcal{D}^{\mathcal{H}}}\eta^{\mathcal{D}^{\mathcal{H}}}_m$
death outside the hospitals	$d^C_{k,m}$	$c_{k,m}$	$\Gamma(\mu^{\mathcal{O}}, \xi^{\mathcal{O}}) + \Gamma(\mu^{\mathcal{D}^C}, \xi^{\mathcal{D}^C})$	$\tau^{\mathcal{D}^{\mathcal{C}}}\eta^{\mathcal{D}^{\mathcal{C}}}_m$

time from the previous state to the next one. We model each $\tau^{\mathcal{X}}_{*,m}$ as in (11) and (12) with the exception of $\tau^{\mathcal{H}^O}_{*,m} = \tau^{\mathcal{U}^O}_{*,m} = 1$, since in our data $H^O_{t,m}$ is just time-lagged $H^I_{t,m}$ (this data source does not separate deceased patients from cured). If the previous state $x_{t,m} = c_{t,m}$, then $\pi^{\mathcal{X}} \sim \Gamma(\mu^{\mathcal{O}}, \xi^{\mathcal{O}}) + \Gamma(\mu^{\mathcal{X}}, \xi^{\mathcal{X}})$, where the second one is the distribution of the time from onset to state $\mathcal{X}$. Otherwise, $\pi^{\mathcal{X}} \sim \Gamma(\mu^{\mathcal{X}}, \xi^{\mathcal{X}})$ is the time from the previous state to state $\mathcal{X}$. See Table S1 for explicit definitions of all the elements of the different versions of formula (13). In Table S2, chosen values for the deterministic parameters of the model are given. In Figure S1, we give the graphical presentation of the assumed distributions of times.

We model the expected number of hospitalized patients $h_{t,m}$ and number of patients in ICUs $u_{t,m}$ differently:

$$h_{1,m} = h^I_{1,m} - h^O_{1,m}, \quad h_{t,m} = h_{t-1,m} + h^I_{t,m} - h^O_{t,m},\ t = 2, \dots, n, \tag{14}$$

and similarly for $u_{t,m}$. We use data on $H_{t,m}$ in addition to $H^I_{t,m}$ and $H^O_{t,m}$ due to some inconsistencies in our data sources between the three and we did similar for ICU. This additional modeling was not necessary for deaths as $D_{t,m} = D^C_{t,m} + D^H_{t,m}$ holds for all t and m in our data, so daily number of deaths was forecasted using $d_{t,m} = d^C_{t,m} + d^H_{t,m}$.

Table S2: Chosen values for deterministic parameters, defined in Table S1, where $\mathcal{O}$ parameters were taken as in Flaxman et al. [1, 2] and others were chosen in exploratory fashion.

$\mathcal{X}$	$\mu^{\mathcal{X}}$	$\xi^{\mathcal{X}}$	$\tau^{\mathcal{X}}$
$\mathcal{O}$	5.1	0.86	
$\mathcal{P}$	6	0.25	0.2
$\mathcal{H}^I$	7	0.45	0.2
$\mathcal{H}^O$	14	0.45	
$\mathcal{U}^I$	4	0.45	0.2
$\mathcal{U}^O$	11	0.45	
$\mathcal{D}^H$	7	0.45	0.2
$\mathcal{D}^C$	17	0.45	0.01

S4 Model specification

According to Bayes theorem, the joint posterior distribution $p(\boldsymbol{\theta}|\boldsymbol{\mathcal{D}})$ is proportional to $p(\boldsymbol{\mathcal{D}}|\boldsymbol{\theta})p(\boldsymbol{\theta})$ where $p(\boldsymbol{\mathcal{D}}|\boldsymbol{\theta})$ is the likelihood of the data $\boldsymbol{\mathcal{D}}$ and $p(\boldsymbol{\theta})$ is the joint prior distribution for all the model parameters $\boldsymbol{\theta}$. Specifically, the vector of parameters is

$$\begin{aligned}\boldsymbol{\theta} = (&\{\{c_{t,m}, R_{t,m}\}_{t=1}^{n_m}\}_{m=1}^2, \{R_{0,m}\}_{m=1}^2, \{\beta_k\}_{k=1}^3, \{\gamma_m\}_{m=1}^2, \{\{\alpha_{l,m}\}_{l=1}^L\}_{m=1}^2, \kappa, \nu,\\ &\{\{p_{t,m}, h_{t,m}, h_{t,m}^I, h_{t,m}^O, u_{t,m}, u_{t,m}^I, u_{t,m}^O, d_{t,m}^H, d_{t,m}^C\}_{t=1}^{n_m}\}_{m=1}^2,\\ &\{\phi^{\mathcal{X}}, \psi^{\mathcal{X}}, \{\tau_{*,m}^{\mathcal{X}}, \eta_m^{\mathcal{X}}\}_{m=1}^2\}_{\mathcal{X}}),\end{aligned}$$

where $\mathcal{X}$ goes through all data sources, i.e. $\mathcal{P}$ (positive cases), $\mathcal{H}$ (hospital), $\mathcal{H}^I$ (hospital in), $\mathcal{H}^O$ (hospital out), $\mathcal{U}$ (ICU), $\mathcal{U}^I$ (ICU in), $\mathcal{U}^O$ (ICU out), $\mathcal{D}^H$ (death in hospitals), $\mathcal{D}^C$ (death outside hospitals). The data are

$$\boldsymbol{\mathcal{D}} = \left(\{\{P_{t,m}, H_{t,m}, H_{t,m}^I, H_{t,m}^O, U_{t,m}, U_{t,m}^I, U_{t,m}^O, D_{t,m}^H, D_{t,m}^C\}_{t=1}^{n_m}\}_{m=1}^2\right).$$

We assume conditional independence of different data sources and waves given the model parameters, so the likelihood can be expressed as

$$p(\boldsymbol{\mathcal{D}}|\boldsymbol{\theta}) = \prod_{m=1}^2 \left(\prod_{t=1}^{n_m} p(P_{t,m}|p_{t,m}, \phi^{\mathcal{P}}) \cdot \ldots \cdot \prod_{t=1}^{n_m} p(D_{t,m}^C|d_{t,m}^C, \phi^{\mathcal{D}^C})\right),$$

where $p(P_{t,m}|p_{t,m}, \phi^{\mathcal{P}})$ is the probability density function of $\text{NegBin}(p_{t,m}, p_{t,m} + p_{t,m}^2/\phi^{\mathcal{P}})$ as specified in (8), similarly for others.

We assume independent prior distributions if not stated otherwise in the previous subsections. In particular, the joint prior distribution is

$$p(\boldsymbol{\theta}) = \left(\prod_{m=1}^{2} p(R_{0,m}) p(\gamma_m) \left(\prod_{l=1}^{L} p(\alpha_{l,m}|\kappa) \right) p(c_{1,m}|\nu) \right) p(\kappa) p(\nu) \left(\prod_{k=1}^{3} p(\beta_k) \right)$$
$$\left(\prod_{\mathcal{X}} p(\phi^{\mathcal{X}}|\psi^{\mathcal{X}}) p(\psi^{\mathcal{X}}) \prod_{m=1}^{2} p(\eta_m^{\mathcal{X}}) \right)$$

where the distributions of priors are specified in (4), (5), (6), (7), (10) and (12), whereas all other parameters of $\boldsymbol{\theta}$ that are not specified in the above joint prior are transformed parameters via (2), (3), (9), (11), (13) and (14).

In addition to the posterior distributions for all the parameters, including the expected numbers of individuals at all states ($x_{t,m}$), the outputs of our model are also their forecasts for a specified number of days based on the posterior predictive distribution (the reliability of forecasts is validated using methods presented in section Computational aspects and model validation in the paper).

References

[1] S. Flaxman, S. Mishra, A. Gandy, H. Unwin, T. Mellan, H. Coupland, C. Whittaker, H. Zhu, T. Berah, J. Eaton, M. Monod, A. Ghani, C. Donnelly, S. Riley, M. Vollmer, N. Ferguson, L. Okell, S. Bhatt, P. Perez-Guzman, P. Walker, Estimating the effects of non-pharmaceutical interventions on covid-19 in europe, Nature 584 (2020) 257–261. doi:10.1038/s41586-020-2405-7.

[2] D. Manevski, N. Ružić Gorenjec, N. Kejžar, R. Blagus, Modeling covid-19 pandemic using bayesian analysis with application to slovene data, Mathematical Biosciences 329 (2020) 108466. doi:https://doi.org/10.1016/j.mbs.2020.108466.
URL http://www.sciencedirect.com/science/article/pii/S0025556420301279
