# Supplementary material for: COVID-19 in Slovenia, from a Success Story to Disaster: What Lessons Can Be Learned?
Source: Life (Basel). 2021 Oct 4;11(10):1045. doi: 10.3390/life11101045 (PMC8541564; doi:10.3390/life11101045)
Supplement: Supplementary file 1 [file life-11-01045-s001.zip › S3_file.html]

 

 

 

 
 
 


 


 COVID-19 in Slovenia, from a success story to disaster: what lessons can be learned? Supplementary material: complete results 

 
 
 
 
 
 
 
 
 

 
 
 


 


 


 

 

 


 

 


 


 


 COVID-19 in Slovenia, from a success story to disaster: what lessons can be learned? Supplementary material: complete results 
 Nina Ružić Gorenjec, Nataša Kejžar, Damjan Manevski, Maja Pohar Perme, Bor Vratanar, Rok Blagus 

 


 
  
 
 Summary 
 
 DF=5 
 
 Model with mobility 
 The following table shows posterior means [with 90 % credible intervals - CI], or true data for quantities ending with “d”. Abbreviations beginning with T as total are expressed as numbers per 1 million inhabitants per 14 days: cases (TC), positive tests (TP), deaths (TD), deaths in hospitals (TDH), deaths not occurring in hospitals (TDC), admissions to hospitals (TH), and admissions to ICUs (TU). The ratios correspond to periods in columns and are in percentages (%). 
 
 
 
  
 Feb 28 - June 30 
 July 1 - Dec 1 
 July 1 - Oct 25 
 Oct 26 - Dec 1 
 
 
 
 
 TC 
 298 [207-486] 
 14727 [9616-27778] 
 6962 [4559-12901] 
 39405 [25498-75273] 
 
 
 TP 
 76 [67-86] 
 3474 [3219-3768] 
 1042 [967-1130] 
 11156 [10321-12118] 
 
 
 TPd 
 88 
 3410 
 1295 
 10096 
 
 
 TD 
 6 [5-8] 
 53 [49-57] 
 11 [10-12] 
 186 [172-199] 
 
 
 TDd 
 6 
 60 
 7 
 227 
 
 
 TDH 
 3 [3-4] 
 39 [36-43] 
 8 [8-9] 
 137 [125-149] 
 
 
 TDHd 
 3 
 44 
 6 
 167 
 
 
 TDC 
 3 [3-4] 
 14 [12-16] 
 3 [3-3] 
 49 [43-55] 
 
 
 TDCd 
 3 
 14 
 2 
 55 
 
 
 TH 
 21 [20-22] 
 191 [185-197] 
 55 [54-57] 
 618 [598-642] 
 
 
 THd 
 20 
 242 
 69 
 790 
 
 
 TU 
 6 [6-7] 
 37 [36-38] 
 9 [8-9] 
 125 [121-129] 
 
 
 TUd 
 4 
 36 
 10 
 119 
 
 
 TP/TC 
 25.68 [15.48-36.26] 
 23.61 [12.39-35.74] 
 15.04 [7.97-22.7] 
 28.31 [14.9-43.49] 
 
 
 TD/TC 
 2.17 [1.34-3.18] 
 0.36 [0.19-0.56] 
 0.16 [0.09-0.25] 
 0.47 [0.24-0.74] 
 
 
 TDH/TC 
 1.06 [0.62-1.64] 
 0.27 [0.14-0.41] 
 0.12 [0.06-0.18] 
 0.35 [0.18-0.55] 
 
 
 TDH/TH 
 15.14 [11.98-18.62] 
 20.62 [18.73-22.53] 
 15.17 [13.78-16.59] 
 22.16 [20.13-24.23] 
 
 
 TDC/TC 
 1.1 [0.67-1.61] 
 0.09 [0.05-0.15] 
 0.02 [0.01-0.03] 
 0.33 [0.17-0.51] 
 
 
 TH/TC 
 7.11 [4.32-10.13] 
 1.29 [0.68-1.98] 
 0.38 [0.2-0.58] 
 4.2 [2.22-6.42] 
 
 
 TU/TC 
 2.16 [1.31-3.11] 
 0.25 [0.13-0.38] 
 0.13 [0.07-0.19] 
 0.32 [0.17-0.49] 
 
 
 TU/TH 
 30.51 [28.68-32.38] 
 19.26 [18.51-20.06] 
 15.85 [15.24-16.51] 
 20.22 [19.42-21.06] 
 
 
 
 
 
 Model without mobility 
 The following table shows posterior means [with 90 % credible intervals - CI], or true data for quantities ending with “d”. Abbreviations beginning with T as total are expressed as numbers per 1 million inhabitants per 14 days: cases (TC), positive tests (TP), deaths (TD), deaths in hospitals (TDH), deaths not occurring in hospitals (TDC), admissions to hospitals (TH), and admissions to ICUs (TU). The ratios correspond to periods in columns and are in percentages (%). 
 
 
 
  
 Feb 28 - June 30 
 July 1 - Dec 1 
 July 1 - Oct 25 
 Oct 26 - Dec 1 
 
 
 
 
 TC 
 287 [201-459] 
 19010 [11608-47658] 
 7889 [4763-20520] 
 54220 [32582-136065] 
 
 
 TP 
 76 [67-87] 
 3506 [3247-3815] 
 1017 [942-1104] 
 11380 [10506-12404] 
 
 
 TPd 
 88 
 3410 
 1295 
 10096 
 
 
 TD 
 7 [6-8] 
 53 [49-57] 
 11 [10-12] 
 185 [171-199] 
 
 
 TDd 
 6 
 60 
 7 
 227 
 
 
 TDH 
 3 [3-4] 
 39 [36-43] 
 8 [8-9] 
 137 [125-149] 
 
 
 TDHd 
 3 
 44 
 6 
 167 
 
 
 TDC 
 3 [3-4] 
 14 [12-16] 
 3 [3-3] 
 49 [43-55] 
 
 
 TDCd 
 3 
 14 
 2 
 55 
 
 
 TH 
 21 [20-22] 
 193 [187-199] 
 54 [52-56] 
 631 [608-656] 
 
 
 THd 
 20 
 242 
 69 
 790 
 
 
 TU 
 6 [6-7] 
 37 [35-38] 
 9 [8-9] 
 125 [121-130] 
 
 
 TUd 
 4 
 36 
 10 
 119 
 
 
 TP/TC 
 26.67 [16.85-37.83] 
 18.58 [7.34-29.95] 
 13 [5.02-20.96] 
 21.12 [8.45-34.52] 
 
 
 TD/TC 
 2.28 [1.42-3.24] 
 0.28 [0.11-0.46] 
 0.14 [0.06-0.24] 
 0.34 [0.14-0.57] 
 
 
 TDH/TC 
 1.11 [0.67-1.69] 
 0.21 [0.08-0.34] 
 0.11 [0.04-0.18] 
 0.25 [0.1-0.42] 
 
 
 TDH/TH 
 15.18 [11.96-18.73] 
 20.37 [18.51-22.24] 
 15.54 [14.11-16.97] 
 21.66 [19.71-23.67] 
 
 
 TDC/TC 
 1.14 [0.72-1.66] 
 0.07 [0.03-0.12] 
 0.02 [0.01-0.03] 
 0.25 [0.1-0.43] 
 
 
 TH/TC 
 7.42 [4.63-10.58] 
 1.01 [0.4-1.67] 
 0.28 [0.11-0.47] 
 3.33 [1.32-5.46] 
 
 
 TU/TC 
 2.26 [1.42-3.22] 
 0.19 [0.08-0.32] 
 0.11 [0.04-0.18] 
 0.23 [0.09-0.39] 
 
 
 TU/TH 
 30.48 [28.56-32.54] 
 19.02 [18.23-19.88] 
 16.03 [15.36-16.78] 
 19.83 [18.99-20.74] 
 
 
 
 
 
 
 DF=4 
 
 Model with mobility 
 The following table shows posterior means [with 90 % credible intervals - CI], or true data for quantities ending with “d”. Abbreviations beginning with T as total are expressed as numbers per 1 million inhabitants per 14 days: cases (TC), positive tests (TP), deaths (TD), deaths in hospitals (TDH), deaths not occurring in hospitals (TDC), admissions to hospitals (TH), and admissions to ICUs (TU). The ratios correspond to periods in columns and are in percentages (%). 
 
 
 
  
 Feb 28 - June 30 
 July 1 - Dec 1 
 July 1 - Oct 25 
 Oct 26 - Dec 1 
 
 
 
 
 TC 
 323 [223-530] 
 13497 [9234-23431] 
 6566 [4488-11365] 
 35408 [24303-61393] 
 
 
 TP 
 75 [66-85] 
 3449 [3211-3729] 
 1044 [971-1130] 
 11050 [10267-11994] 
 
 
 TPd 
 88 
 3410 
 1295 
 10096 
 
 
 TD 
 6 [5-8] 
 53 [50-57] 
 11 [10-12] 
 186 [173-199] 
 
 
 TDd 
 6 
 60 
 7 
 227 
 
 
 TDH 
 3 [3-4] 
 39 [36-43] 
 8 [8-9] 
 137 [126-149] 
 
 
 TDHd 
 3 
 44 
 6 
 167 
 
 
 TDC 
 3 [3-4] 
 14 [12-16] 
 3 [3-3] 
 49 [43-55] 
 
 
 TDCd 
 3 
 14 
 2 
 55 
 
 
 TH 
 21 [20-22] 
 190 [185-196] 
 56 [54-57] 
 616 [597-639] 
 
 
 THd 
 20 
 242 
 69 
 790 
 
 
 TU 
 6 [6-7] 
 37 [36-38] 
 9 [8-9] 
 125 [121-129] 
 
 
 TUd 
 4 
 36 
 10 
 119 
 
 
 TP/TC 
 23.24 [14.19-33.43] 
 25.55 [15.07-37.17] 
 15.93 [9.35-23.26] 
 31.17 [18.32-45.47] 
 
 
 TD/TC 
 1.99 [1.21-2.89] 
 0.39 [0.22-0.58] 
 0.17 [0.1-0.25] 
 0.52 [0.3-0.77] 
 
 
 TDH/TC 
 0.97 [0.57-1.5] 
 0.29 [0.17-0.43] 
 0.13 [0.07-0.19] 
 0.38 [0.22-0.57] 
 
 
 TDH/TH 
 15.07 [11.95-18.57] 
 20.68 [18.86-22.5] 
 15.07 [13.74-16.44] 
 22.27 [20.3-24.25] 
 
 
 TDC/TC 
 1 [0.61-1.48] 
 0.1 [0.06-0.15] 
 0.02 [0.01-0.03] 
 0.36 [0.21-0.53] 
 
 
 TH/TC 
 6.49 [3.95-9.39] 
 1.41 [0.81-2.06] 
 0.41 [0.24-0.6] 
 4.56 [2.63-6.66] 
 
 
 TU/TC 
 1.96 [1.2-2.87] 
 0.27 [0.16-0.4] 
 0.13 [0.08-0.2] 
 0.35 [0.2-0.52] 
 
 
 TU/TH 
 30.39 [28.52-32.35] 
 19.29 [18.54-20.06] 
 15.8 [15.15-16.44] 
 20.29 [19.49-21.11] 
 
 
 
 
 
 Model without mobility 
 The following table shows posterior means [with 90 % credible intervals - CI], or true data for quantities ending with “d”. Abbreviations beginning with T as total are expressed as numbers per 1 million inhabitants per 14 days: cases (TC), positive tests (TP), deaths (TD), deaths in hospitals (TDH), deaths not occurring in hospitals (TDC), admissions to hospitals (TH), and admissions to ICUs (TU). The ratios correspond to periods in columns and are in percentages (%). 
 
 
 
  
 Feb 28 - June 30 
 July 1 - Dec 1 
 July 1 - Oct 25 
 Oct 26 - Dec 1 
 
 
 
 
 TC 
 308 [219-488] 
 15915 [9587-37623] 
 7982 [4829-18754] 
 41084 [24583-96939] 
 
 
 TP 
 75 [66-86] 
 3456 [3200-3747] 
 1022 [946-1109] 
 11150 [10305-12117] 
 
 
 TPd 
 88 
 3410 
 1295 
 10096 
 
 
 TD 
 6 [6-8] 
 53 [50-57] 
 12 [11-12] 
 186 [172-199] 
 
 
 TDd 
 6 
 60 
 7 
 227 
 
 
 TDH 
 3 [3-4] 
 39 [36-43] 
 9 [8-9] 
 137 [125-149] 
 
 
 TDHd 
 3 
 44 
 6 
 167 
 
 
 TDC 
 3 [3-4] 
 14 [12-16] 
 3 [3-3] 
 48 [43-55] 
 
 
 TDCd 
 3 
 14 
 2 
 55 
 
 
 TH 
 21 [20-22] 
 190 [184-197] 
 55 [53-57] 
 620 [596-645] 
 
 
 THd 
 20 
 242 
 69 
 790 
 
 
 TU 
 6 [6-7] 
 37 [35-38] 
 9 [8-9] 
 125 [120-130] 
 
 
 TUd 
 4 
 36 
 10 
 119 
 
 
 TP/TC 
 24.34 [15.55-34.5] 
 21.79 [9.21-35.68] 
 12.84 [5.44-21.02] 
 27.33 [11.45-44.87] 
 
 
 TD/TC 
 2.12 [1.33-2.99] 
 0.34 [0.14-0.56] 
 0.14 [0.06-0.24] 
 0.45 [0.19-0.76] 
 
 
 TDH/TC 
 1.04 [0.62-1.57] 
 0.25 [0.1-0.41] 
 0.11 [0.05-0.18] 
 0.33 [0.14-0.56] 
 
 
 TDH/TH 
 15.02 [11.93-18.74] 
 20.76 [18.86-22.59] 
 15.78 [14.31-17.17] 
 22.14 [20.1-24.1] 
 
 
 TDC/TC 
 1.06 [0.67-1.51] 
 0.09 [0.04-0.15] 
 0.02 [0.01-0.03] 
 0.3 [0.13-0.52] 
 
 
 TH/TC 
 6.94 [4.34-9.76] 
 1.2 [0.51-1.98] 
 0.34 [0.15-0.57] 
 3.89 [1.65-6.43] 
 
 
 TU/TC 
 2.1 [1.32-2.96] 
 0.23 [0.1-0.38] 
 0.11 [0.05-0.18] 
 0.3 [0.13-0.51] 
 
 
 TU/TH 
 30.3 [28.46-32.28] 
 19.29 [18.47-20.15] 
 16.21 [15.52-16.93] 
 20.15 [19.28-21.06] 
 
 
 
 
 
 
 DF=3 
 
 Model with mobility 
 The following table shows posterior means [with 90 % credible intervals - CI], or true data for quantities ending with “d”. Abbreviations beginning with T as total are expressed as numbers per 1 million inhabitants per 14 days: cases (TC), positive tests (TP), deaths (TD), deaths in hospitals (TDH), deaths not occurring in hospitals (TDC), admissions to hospitals (TH), and admissions to ICUs (TU). The ratios correspond to periods in columns and are in percentages (%). 
 
 
 
  
 Feb 28 - June 30 
 July 1 - Dec 1 
 July 1 - Oct 25 
 Oct 26 - Dec 1 
 
 
 
 
 TC 
 399 [265-678] 
 13043 [8822-22907] 
 6432 [4358-11239] 
 34122 [22907-60156] 
 
 
 TP 
 73 [65-83] 
 3439 [3193-3713] 
 1041 [965-1122] 
 11026 [10203-11918] 
 
 
 TPd 
 88 
 3410 
 1295 
 10096 
 
 
 TD 
 6 [5-7] 
 53 [49-57] 
 11 [10-12] 
 186 [172-199] 
 
 
 TDd 
 6 
 60 
 7 
 227 
 
 
 TDH 
 3 [2-4] 
 39 [36-43] 
 8 [8-9] 
 137 [125-150] 
 
 
 TDHd 
 3 
 44 
 6 
 167 
 
 
 TDC 
 3 [3-4] 
 14 [12-16] 
 3 [3-3] 
 49 [43-55] 
 
 
 TDCd 
 3 
 14 
 2 
 55 
 
 
 TH 
 21 [20-21] 
 190 [184-196] 
 55 [54-57] 
 615 [595-636] 
 
 
 THd 
 20 
 242 
 69 
 790 
 
 
 TU 
 6 [6-7] 
 37 [36-38] 
 9 [8-9] 
 125 [121-129] 
 
 
 TUd 
 4 
 36 
 10 
 119 
 
 
 TP/TC 
 18.35 [10.94-27.6] 
 26.25 [15.08-38.76] 
 16.19 [9.32-23.83] 
 32.25 [18.47-47.79] 
 
 
 TD/TC 
 1.58 [0.94-2.38] 
 0.41 [0.23-0.6] 
 0.18 [0.1-0.26] 
 0.54 [0.31-0.81] 
 
 
 TDH/TC 
 0.77 [0.44-1.23] 
 0.3 [0.17-0.45] 
 0.13 [0.07-0.19] 
 0.4 [0.23-0.6] 
 
 
 TDH/TH 
 14.98 [11.86-18.64] 
 20.73 [18.88-22.61] 
 15.12 [13.77-16.52] 
 22.33 [20.33-24.37] 
 
 
 TDC/TC 
 0.79 [0.47-1.22] 
 0.11 [0.06-0.16] 
 0.02 [0.01-0.03] 
 0.37 [0.21-0.56] 
 
 
 TH/TC 
 5.17 [3.07-7.76] 
 1.45 [0.82-2.15] 
 0.42 [0.24-0.63] 
 4.7 [2.67-6.97] 
 
 
 TU/TC 
 1.57 [0.91-2.37] 
 0.28 [0.16-0.42] 
 0.14 [0.08-0.2] 
 0.37 [0.21-0.55] 
 
 
 TU/TH 
 30.35 [28.57-32.24] 
 19.35 [18.6-20.11] 
 15.84 [15.2-16.48] 
 20.34 [19.54-21.16] 
 
 
 
 
 
 Model without mobility 
 The following table shows posterior means [with 90 % credible intervals - CI], or true data for quantities ending with “d”. Abbreviations beginning with T as total are expressed as numbers per 1 million inhabitants per 14 days: cases (TC), positive tests (TP), deaths (TD), deaths in hospitals (TDH), deaths not occurring in hospitals (TDC), admissions to hospitals (TH), and admissions to ICUs (TU). The ratios correspond to periods in columns and are in percentages (%). 
 
 
 
  
 Feb 28 - June 30 
 July 1 - Dec 1 
 July 1 - Oct 25 
 Oct 26 - Dec 1 
 
 
 
 
 TC 
 353 [245-571] 
 12135 [8328-20907] 
 6278 [4282-10750] 
 30799 [21028-53494] 
 
 
 TP 
 74 [65-84] 
 3422 [3176-3708] 
 1031 [956-1116] 
 10985 [10182-11942] 
 
 
 TPd 
 88 
 3410 
 1295 
 10096 
 
 
 TD 
 6 [5-8] 
 53 [50-57] 
 12 [11-13] 
 186 [172-199] 
 
 
 TDd 
 6 
 60 
 7 
 227 
 
 
 TDH 
 3 [3-4] 
 40 [36-43] 
 9 [8-9] 
 137 [125-149] 
 
 
 TDHd 
 3 
 44 
 6 
 167 
 
 
 TDC 
 3 [3-4] 
 14 [12-16] 
 3 [3-3] 
 49 [43-55] 
 
 
 TDCd 
 3 
 14 
 2 
 55 
 
 
 TH 
 21 [20-22] 
 188 [182-194] 
 55 [53-57] 
 609 [587-631] 
 
 
 THd 
 20 
 242 
 69 
 790 
 
 
 TU 
 6 [6-7] 
 37 [36-38] 
 9 [9-9] 
 124 [120-129] 
 
 
 TUd 
 4 
 36 
 10 
 119 
 
 
 TP/TC 
 20.95 [12.87-29.87] 
 28.13 [16.57-40.93] 
 16.41 [9.68-23.95] 
 35.64 [20.95-52] 
 
 
 TD/TC 
 1.81 [1.11-2.6] 
 0.44 [0.25-0.65] 
 0.19 [0.11-0.28] 
 0.6 [0.35-0.9] 
 
 
 TDH/TC 
 0.89 [0.52-1.37] 
 0.32 [0.19-0.48] 
 0.14 [0.08-0.21] 
 0.44 [0.26-0.66] 
 
 
 TDH/TH 
 14.97 [12.06-18.44] 
 21.04 [19.18-22.96] 
 15.86 [14.42-17.33] 
 22.52 [20.54-24.57] 
 
 
 TDC/TC 
 0.92 [0.56-1.33] 
 0.11 [0.07-0.17] 
 0.02 [0.01-0.04] 
 0.4 [0.23-0.6] 
 
 
 TH/TC 
 5.95 [3.69-8.55] 
 1.54 [0.89-2.25] 
 0.45 [0.26-0.66] 
 5 [2.9-7.32] 
 
 
 TU/TC 
 1.8 [1.11-2.61] 
 0.3 [0.17-0.44] 
 0.14 [0.08-0.21] 
 0.4 [0.23-0.59] 
 
 
 TU/TH 
 30.29 [28.39-32.26] 
 19.56 [18.74-20.41] 
 16.39 [15.68-17.12] 
 20.46 [19.6-21.35] 
 
 
 
 
 
 
 
 First wave 
 
 DF=5 
 
 Model with mobility 
    
 
 
 Model without mobility 
    
 
 
 
 DF=4 
 
 Model with mobility 
    
 
 
 Model without mobility 
    
 
 
 
 DF=3 
 
 Model with mobility 
    
 
 
 Model without mobility 
    
 
 
 
 
 Second wave 
 
 DF=5 
 
 Model with mobility 
    
 
 
 Model with mobility 
    
 
 
 
 DF=4 
 
 Model with mobility 
    
 
 
 Model without mobility 
    
 
 
 
 DF=3 
 
 Model with mobility 
    
 
 
 Model with mobility 
    
 
 
 
 
 Summary of counterfactual analysis 
 
 DF=5 
    
 
 
 
  
 Scenario 
 Contact-tracing 
 Cumulative deaths 
 Hospitalizations 
 ICU 
 
 
 
 
 2 
 (1) 
 (a) 
 1217 [1128 to 1303] 
 1338 [1256 to 1430] 
 223 [212 to 234] 
 
 
 3 
 (1) 
 (b) 
 308 [266 to 369] 
 281 [245 to 322] 
 48 [41 to 55] 
 
 
 4 
 (2) 
 (a) 
 9985 [5216 to 15545] 
 9654 [3366 to 17871] 
 1647 [561 to 3066] 
 
 
 5 
 (2) 
 (b) 
 6306 [3349 to 10261] 
 5260 [2562 to 8664] 
 888 [434 to 1461] 
 
 
 6 
 (3) 
 (a) 
 3 [2 to 4] 
 6 [4 to 7] 
 1 [1 to 1] 
 
 
 7 
 (3) 
 (b) 
 3 [2 to 4] 
 6 [4 to 7] 
 1 [1 to 1] 
 
 
 8 
 (4) 
 (a) 
 842 [768 to 923] 
 983 [940 to 1029] 
 170 [162 to 177] 
 
 
 9 
 (4) 
 (b) 
 297 [261 to 348] 
 281 [245 to 322] 
 48 [41 to 55] 
 
 
 10 
 (5) 
 (a) 
 242 [168 to 359] 
 169 [113 to 271] 
 29 [19 to 46] 
 
 
 11 
 (5) 
 (b) 
 204 [147 to 296] 
 169 [113 to 271] 
 29 [19 to 46] 
 
 
 12 
 (6) 
 (a) 
 37 [28 to 53] 
 35 [28 to 46] 
 6 [5 to 8] 
 
 
 13 
 (6) 
 (b) 
 36 [28 to 50] 
 35 [28 to 46] 
 6 [5 to 8] 
 
 
 14 
 (5.1) 
 (a) 
 103 [82 to 151] 
 66 [62 to 70] 
 11 [11 to 12] 
 
 
 15 
 (5.1) 
 (b) 
 83 [73 to 98] 
 66 [62 to 70] 
 11 [11 to 12] 
 
 
 16 
 (6.1) 
 (a) 
 24 [21 to 31] 
 20 [19 to 21] 
 3 [3 to 4] 
 
 
 17 
 (6.1) 
 (b) 
 24 [21 to 29] 
 20 [19 to 21] 
 3 [3 to 4] 
 
 
 18 
 (7) 
 (a) 
 169 [148 to 207] 
 160 [151 to 170] 
 27 [26 to 29] 
 
 
 19 
 (7) 
 (b) 
 154 [140 to 170] 
 160 [152 to 169] 
 27 [26 to 29] 
 
 
 
 
 
 
 
 
 
 
 
 
 
 
 
 
 
 
 
 
 
 
 
 
 
 
 
 
 
 
 
 
 
 
 
 
 
 
 
 
 
 
 DF=4 
    
 
 
 
  
 Scenario 
 Contact-tracing 
 Cumulative deaths 
 Hospitalizations 
 ICU 
 
 
 
 
 2 
 (1) 
 (a) 
 1217 [1132 to 1304] 
 1341 [1292 to 1398] 
 224 [214 to 233] 
 
 
 3 
 (1) 
 (b) 
 324 [281 to 383] 
 296 [258 to 343] 
 50 [44 to 58] 
 
 
 4 
 (2) 
 (a) 
 10711 [5977 to 16080] 
 8231 [2617 to 16175] 
 1376 [428 to 2773] 
 
 
 5 
 (2) 
 (b) 
 8017 [4683 to 11956] 
 6513 [3494 to 9891] 
 1091 [586 to 1677] 
 
 
 6 
 (3) 
 (a) 
 3 [2 to 4] 
 6 [4 to 7] 
 1 [1 to 1] 
 
 
 7 
 (3) 
 (b) 
 3 [2 to 4] 
 6 [4 to 7] 
 1 [1 to 1] 
 
 
 8 
 (4) 
 (a) 
 812 [753 to 875] 
 971 [940 to 1006] 
 167 [161 to 173] 
 
 
 9 
 (4) 
 (b) 
 309 [272 to 357] 
 296 [258 to 343] 
 50 [44 to 58] 
 
 
 10 
 (5) 
 (a) 
 269 [188 to 399] 
 227 [152 to 354] 
 38 [25 to 61] 
 
 
 11 
 (5) 
 (b) 
 246 [175 to 360] 
 227 [152 to 354] 
 38 [25 to 61] 
 
 
 12 
 (6) 
 (a) 
 38 [29 to 52] 
 40 [31 to 52] 
 7 [5 to 9] 
 
 
 13 
 (6) 
 (b) 
 38 [29 to 51] 
 40 [31 to 52] 
 7 [5 to 9] 
 
 
 14 
 (5.1) 
 (a) 
 89 [77 to 109] 
 65 [62 to 68] 
 11 [10 to 12] 
 
 
 15 
 (5.1) 
 (b) 
 78 [71 to 87] 
 65 [62 to 68] 
 11 [10 to 12] 
 
 
 16 
 (6.1) 
 (a) 
 23 [20 to 26] 
 20 [18 to 21] 
 3 [3 to 4] 
 
 
 17 
 (6.1) 
 (b) 
 22 [20 to 25] 
 20 [18 to 21] 
 3 [3 to 4] 
 
 
 18 
 (7) 
 (a) 
 155 [141 to 173] 
 155 [148 to 164] 
 27 [25 to 28] 
 
 
 19 
 (7) 
 (b) 
 147 [136 to 161] 
 155 [148 to 164] 
 27 [25 to 28] 
 
 
 
 
 
 
 
 
 
 
 
 
 
 
 
 
 
 
 
 
 
 
 
 
 
 
 
 
 
 
 
 
 
 
 
 
 
 
 
 
 
 
 DF=3 
    
 
 
 
  
 Scenario 
 Contact-tracing 
 Cumulative deaths 
 Hospitalizations 
 ICU 
 
 
 
 
 2 
 (1) 
 (a) 
 1218 [1130 to 1306] 
 1344 [1294 to 1396] 
 224 [215 to 234] 
 
 
 3 
 (1) 
 (b) 
 346 [302 to 404] 
 315 [278 to 358] 
 53 [47 to 61] 
 
 
 4 
 (2) 
 (a) 
 10374 [5729 to 15816] 
 7342 [3901 to 11306] 
 1232 [671 to 1931] 
 
 
 5 
 (2) 
 (b) 
 8662 [5077 to 12800] 
 7342 [3901 to 11306] 
 1232 [671 to 1931] 
 
 
 6 
 (3) 
 (a) 
 3 [2 to 4] 
 6 [4 to 7] 
 1 [1 to 1] 
 
 
 7 
 (3) 
 (b) 
 3 [2 to 4] 
 6 [4 to 7] 
 1 [1 to 1] 
 
 
 8 
 (4) 
 (a) 
 823 [761 to 888] 
 972 [941 to 1006] 
 168 [162 to 174] 
 
 
 9 
 (4) 
 (b) 
 328 [290 to 375] 
 315 [278 to 358] 
 53 [47 to 61] 
 
 
 10 
 (5) 
 (a) 
 379 [263 to 564] 
 304 [210 to 438] 
 52 [35 to 75] 
 
 
 11 
 (5) 
 (b) 
 330 [236 to 459] 
 304 [210 to 438] 
 52 [35 to 75] 
 
 
 12 
 (6) 
 (a) 
 51 [37 to 80] 
 49 [38 to 64] 
 8 [6 to 11] 
 
 
 13 
 (6) 
 (b) 
 50 [37 to 74] 
 49 [38 to 64] 
 8 [6 to 11] 
 
 
 14 
 (5.1) 
 (a) 
 100 [84 to 131] 
 65 [62 to 69] 
 11 [11 to 12] 
 
 
 15 
 (5.1) 
 (b) 
 82 [73 to 95] 
 65 [62 to 69] 
 11 [11 to 12] 
 
 
 16 
 (6.1) 
 (a) 
 24 [21 to 31] 
 20 [18 to 21] 
 3 [3 to 3] 
 
 
 17 
 (6.1) 
 (b) 
 24 [21 to 29] 
 20 [18 to 21] 
 3 [3 to 3] 
 
 
 18 
 (7) 
 (a) 
 154 [141 to 169] 
 155 [148 to 161] 
 27 [25 to 28] 
 
 
 19 
 (7) 
 (b) 
 148 [136 to 160] 
 155 [148 to 161] 
 27 [25 to 28] 
 
 
 
 
 
 
 
 
 
 
 
 
 
 
 
 
 
 
 
 
 
 
 
 
 
 
 
 
 
 
 
 
 
 
 
 
 
 
 
 
 
 
 
 
 
 
 
 
 
 
 
 
 
 
 
 
 
 
 
 
 
 
 
 
 
 
 
 
 
 
 
 
 
 
 
 
 
 
 
 
 
 
 
 
 
 
 
 
 
 
 
 
 
 
 
 
 
 
 
 
 
 
 
 
 
 
 
 
 
 
 
 
 
 
 
 
 
 
 
 
 
 
 
 
 
 
 
 
 
 
 
 
 
 
 
 
 
 
 
 
 
 
 
 
 
 
 
 
 
 
 
 
 
 
 
 
 
 
 
 
 
 
 
 
 
 
 
 
 
 
 
 
 
 
 
 
 
 
 
 
 
 
 
 
 
 
 
 
 
 
 
 
 
 
 
 
 
 
 
 
 
 
 
 
 
 
 
 
 
 
 
 
 
 
 
 
 
 
 
 
 
 
 
 
 
 
 
 
 
 
 
 
 
 
 
 
 
 
 
 
 
 
 
 
 
 
 
 
 
 
 
 
 
 
 
 
 
 
 
 
 
 
 
 
 
 
 
 
 
 
 
 
 
 
 
 
 
 
 
 
 
 
 
 
 
 
 
 
 
 
 
 
 
 
 
 
 
 
 
 
 
 
 
 
 
 
 
 
 
 
 
 
 
 
 Counterfactual analysis ( \(\tau\) ,  \(\Delta\) ) 
 
 DF=5 
    
 
 
 DF=4 
    
 
 
 DF=3 
    
 
 
 
 Model without spline 
 
 First wave 
    
 
 
 Second wave 
    
 
 
 Convergence 
    
 
 
 
 Model without contact tracing 
 
 First wave 
 
 DF=5 
 
 Model with mobility 
    
 
 
 Model without mobility 
    
 
 
 
 DF=4 
 
 Model with mobility 
    
 
 
 Model without mobility 
    
 
 
 
 DF=3 
 
 Model with mobility 
    
 
 
 Model without mobility 
    
 
 
 
 
 Second wave 
 
 DF=5 
 
 Model with mobility 
    
 
 
 Model without mobility 
    
 
 
 
 DF=4 
 
 Model with mobility 
    
 
 
 Model without mobility 
    
 
 
 
 DF=3 
 
 Model with mobility 
    
 
 
 Model without mobility 
    
 
 
 
 
 Convergence 
 
 DF=5 
 
 Model with mobility 
    
 
 
 Model without mobility 
    
 
 
 
 DF=4 
 
 Model with mobility 
    
 
 
 Model without mobility 
    
 
 
 
 DF=3 
 
 Model with mobility 
    
 
 
 Model without mobility 
    
 
 
 
 
 
 Convergence 
 
 DF=5 
 
 Model with mobility 
    
 
 
 Model without mobility 
    
 
 
 
 DF=4 
 
 Model with mobility 
    
 
 
 Model without mobility 
    
 
 
 
 DF=3 
 
 Model with mobility 
    
 
 
 Model without mobility 
    
 
 
 
 


 

 

 

 

 


 
 

 
 
