# Supplementary material for: COVID-19 in Slovenia, from a Success Story to Disaster: What Lessons Can Be Learned?
Source: Life (Basel). 2021 Oct 4;11(10):1045. doi: 10.3390/life11101045 (PMC8541564; doi:10.3390/life11101045)
Supplement: Supplementary file 1 [file life-11-01045-s001.zip › Table_S1.html]

Non-parmaceutical interventions in Slovenia during 1st and 2nd wave of coronavirus


# Non-parmaceutical interventions in Slovenia during 1st and 2nd wave of coronavirus

#### from https://covid-19.sledilnik.org/

Major interventions are marked with pink color.

| Date | Non-pharmaceutical intervention (NPI) | Source |
| --- | --- | --- |
| 2020-03-04,Wed | First case | https://www.gov.si/novice/2020-03-04-v-sloveniji-potrjen-prvi-primer-okuzbe-z-novim-koronavirusom/ |
| 2020-03-06,Fri | Prohibition of visits in elderly homes | https://www.gov.si/novice/2020-03-06-popolna-prepoved-obiskov-v-domovih-za-starejse/ |
| 2020-03-07,Sat | Prohibition of events/gatherings of 500 people or more in enclosed spaces | https://www.gov.si/novice/2020-03-07-minister-podpisal-odredbo-o-prepovedi-zbiranja-na-prireditvah-v-javnih-prostorih-s-pojasnilom-o-visini-kazni/ |
| 2020-03-10,Tue | Prohibition of events/gatherings of 100 people or more in enclosed spaces | https://www.gov.si/novice/2020-03-10-v-veljavi-ukrepi-za-zajezitev-sirjenja-koronavirusa/ |
| 2020-03-12,Thu | Proclamation of epidemics | https://www.gov.si/novice/2020-03-12-slovenija-razglasila-epidemijo-novega-koronavirusa/ |
| 2020-03-14,Sat | Testing strategy: hospitalized and health-care workers | https://www.gov.si/novice/2020-03-14-spremenjeno-diagnosticiranje-za-realnejse-nacrtovanje-ukrepov-za-obvladovanje-epidemije/ |
| 2020-03-16,Mon | School and child care closures | https://www.gov.si/novice/2020-03-12-v-sloveniji-bo-razglasena-epidemija-vrtci-in-sole-se-zacasno-zaprejo/ |
| 2020-03-16,Mon | Prohibition of public transport | https://www.gov.si/novice/2020-03-15-vlada-sprejela-odlok-o-zacasni-prepovedi-in-omejitvah-javnega-prevoza-potnikov-v-republiki-sloveniji/ |
| 2020-03-17,Tue | Prohibition of air transport | https://www.gov.si/novice/2020-03-16-prepoved-zracnega-prometa/ |
| 2020-03-18,Wed | Closure of non-vital border-crossings with Croatia | https://www.gov.si/novice/2020-03-18-zaprtje-mejnih-prehodov-med-slovenijo-in-hrvasko/ |
| 2020-03-20,Fri | Prohibition of gatherings in public spaces | https://www.gov.si/novice/2020-03-19-od-polnoci-zacasna-prepoved-zbiranja-ljudi-na-javnih-shodih-prireditvah-in-drugih-dogodkih-na-javnih-krajih/ |
| 2020-03-20,Fri | Testing strategy: hospitalized, health-care workers, people over 60 years, vulnerable groups (invalids, pensioners, pregnant women) | https://www.gov.si/novice/2020-03-22-ministrstvo-za-zdravje-z-vrsto-ukrepov-v-boju-proti-covid-19/ |
| 2020-03-30,Mon | Prohibition of crossing municipality-of-residence borders | https://www.gov.si/novice/2020-03-29-od-polnoci-prepoved-gibanja-izven-obcine-stalnega-ali-zacasnega-prebivalisca/ |
| 2020-03-30,Mon | Mandatory desinfection of apartment buildings | https://www.gov.si/novice/2020-03-29-danes-zacel-veljati-odlok-o-obveznem-razkuzevanju-vecstanovanjskih-stavb/ |
| 2020-03-30,Mon | Shops from 8-10 am oinly for vulnerable groups (invalids, pensioners, pregnant women) | https://www.gov.si/novice/2020-03-29-opravljanje-nakupov-za-ranljive-skupine/ |
| 2020-04-04,Sat | Shops from 8-10 am and last business hour only for vulnerable groups (invalids, pensioners, pregnant women) | https://www.gov.si/novice/2020-04-03-vlada-dopolnila-odlok-o-zacasni-prepovedi-ponujanja-in-prodajanja-blaga-in-storitev-potrosnikom/ |
| 2020-04-08,Wed | Testing strategy: hospitalized, health-care workers, people over 60 years, vulnerable groups (invalids, pensioners, pregnant women), household members | https://www.gov.si/assets/ministrstva/MZ/DOKUMENTI/Koronavirus/145-Dopolnitev-navodil-glede-testiranja-na-COVID-19.pdf |
| 2020-04-12,Sun | Mandatory 7-day quarantine at arrival from abroad | https://www.gov.si/novice/2020-04-11-jutri-zacne-veljati-odlok-o-ukrepih-na-mejnih-prehodih-ob-vstopu-v-republiko-slovenijo/ |
| 2020-04-15,Wed | Testing strategy: everybody (if possible) | https://www.gov.si/assets/ministrstva/MZ/DOKUMENTI/Koronavirus/Druga-dopolnitev-navodil-za-testiranje-na-COVID-19.pdf |
| 2020-04-20,Mon | Release of some business activities (construction, services, outdoor sports ...), some public transport. | https://www.gov.si/novice/2020-04-17-16-april-odlok-o-spremembah-in-dopolnitvah-odloka-o-zacasni-prepovedi-ponujanja-in-prodajanja-blaga-in-storitev-potrosnikom-v-republiki-sloveniji/ |
| 2020-04-21,Tue | Testing strategy: all symptomatic | https://www.gov.si/assets/ministrstva/MZ/DOKUMENTI/Koronavirus/Dodatno-k-Drugi-dopolnitvi-navodil-za-testiranje-na-COVID19-Testiranje-pri-vseh-osebah-s-sumom.pdf |
| 2020-04-25,Sat | Release of some business activities: car wash, insurance | https://www.gov.si/novice/2020-04-24-28-dopisna-seja-vlade-republike-slovenije/ |
| 2020-04-28,Tue | Release of some activities: weddings | https://www.gov.si/novice/2020-04-24-od-torka-28-aprila-2020-ponovno-omogoceno-izvajanje-porok/ |
| 2020-04-29,Wed | Opening of libraries, galeries, museums | https://www.gov.si/novice/2020-04-28-30-dopisna-seja-vlade-republike-slovenije/ |
| 2020-04-30,Thu | Release of municipality border crossing | https://www.gov.si/novice/2020-04-28-30-dopisna-seja-vlade-republike-slovenije/ |
| 2020-05-04,Mon | Release for markets, outdoor food/drink service, shops (up to 400 m2), hairdressers, beauty salons | https://www.gov.si/novice/2020-04-17-16-april-odlok-o-spremembah-in-dopolnitvah-odloka-o-zacasni-prepovedi-ponujanja-in-prodajanja-blaga-in-storitev-potrosnikom-v-republiki-sloveniji/ |
| 2020-05-06,Wed | Release of restrictions for professional sport | https://www.gov.si/novice/2020-05-06-vlada-sprostila-tudi-ukrepe-na-podrocju-vrhunskega-tekmovalnega-sporta/ |
| 2020-05-09,Sat | Release of all health-care and dental services | https://www.gov.si/novice/2020-05-08-vlada-ponovno-uvaja-izvajanje-zdravstvenih-in-zobozdravstvenih-storitev-na-vseh-ravneh/ |
| 2020-05-11,Mon | Release of public transport | https://www.gov.si/novice/2020-05-08-vlada-sprosca-ukrepe-na-podrocju-zdravstvene-dejavnosti-in-javnega-potniskega-prometa-v-sloveniji/ |
| 2020-05-15,Fri | Cancellation of epidemic | https://www.gov.si/novice/2020-05-15-vlada-preklicala-epidemijo-nalezljive-bolezni-sars-cov-2-covid-19/ |
| 2020-05-15,Fri | Release of quarantine (only for foreigners from third countries) | https://www.gov.si/novice/2020-05-15-vlada-spremenila-ukrepe-na-mejnih-prehodih-na-zunanji-meji-in-na-kontrolnih-tockah-na-notranjih-mejah-republike-slovenije/ |
| 2020-05-15,Fri | Release of events/gatherings up to 50 people | https://www.gov.si/novice/2020-05-15-vlada-izdala-odlok-o-zacasni-splosni-omejitvi-zbiranja-ljudi-na-javnih-krajih-in-mestih-v-republiki-sloveniji/ |
| 2020-05-18,Mon | Release of schools: first 1-3rd grade, last year of secondary schools | https://www.gov.si/novice/2020-05-15-preklic-epidemije-nekateri-ucenci-in-dijaki-nazaj-v-sole-z-18-majem/ |
| 2020-05-26,Tue | Mandatory 14-day quarantine for foreigners | https://www.gov.si/novice/2020-05-26-veljati-je-zacel-novi-odlok-o-ukrepih-za-preprecitev-sirjenja-covida-19-na-mejnih-prehodih-in-kontrolnih-tockah/ |
| 2020-06-01,Mon | Release of schools: all | https://www.gov.si/novice/2020-05-28-osnovnosolci-v-sole-1-in-8-junija-srednjesolci-sele-jeseni/ |
| 2020-06-01,Mon | Release of events/gatherings up to 200 | https://www.gov.si/novice/2020-05-28-18-redna-seja-vlade-republike-slovenije/ |
| 2020-06-01,Mon | Release of all services, accomodation facilities | https://www.gov.si/novice/2020-05-28-18-redna-seja-vlade-republike-slovenije/ |
| 2020-06-08,Mon | Release of non-vital border crossings | https://www.gov.si/novice/2020-06-08-od-danes-veljajo-nova-pravila-za-prehajanje-slovenske-meje/ |
| 2020-06-15,Mon | Release of events/gatherings up to 500 people | https://www.gov.si/novice/2020-06-11-20-redna-seja-vlade-republike-slovenije/ |
| 2020-06-30,Tue | Prohibition of events/gatherings of 50 people | https://www.gov.si/novice/2020-06-29-vlada-omejila-zbiranja-ljudi-in-znizala-zneske-trosarin-za-energente/ |
| 2020-07-09,Thu | Prohibition of events/gatherings of 10 people or more | https://www.gov.si/novice/2020-07-08-25-redna-seja-vlade-republike-slovenije/ |
| 2020-08-21,Fri | Mandatory 14-day quarantine comming from Croatia | https://www.gov.si/novice/2020-08-20-28-redna-seja-vlade-republike-slovenije/ |
| 2020-09-10,Thu | Testing strategy: non-mandatory testing for children up to 8 years with symptoms of cold | https://www.24ur.com/novice/korona/otrok-do-konca-prve-triade-z-blagimi-znaki-okuzbe-dihal-ne-bodo-vec-testirali.html |
| 2020-09-19,Sat | Mandatory facial masks in all public enclosed spaces and outside (distance < 2m) | https://www.gov.si/novice/2020-09-18-92-dopisna-seja-vlade-republike-slovenije/ |
| 2020-10-04,Sun | Release of facial masks for primary school pupils | https://www.uradni-list.si/\_pdf/2020/Ur/u2020135.pdf |
| 2020-10-07,Wed | Mandatory dispensers in appartment buildings | https://www.uradni-list.si/\_pdf/2020/Ur/u2020135.pdf |
| 2020-10-09,Fri | Partial contact tracing only (priority in elderly homes, health-care amenities, schools and households) | https://www.sta.si/2816961/zaradi-prevec-okuzb-s-koronavirusom-epidemiologi-dolocili-prioritete-pri-iskanju-stikov |
| 2020-10-09,Fri | Restrictions of activities in enclosed spaces (20m2 per person). | https://www.uradni-list.si/\_pdf/2020/Ur/u2020138.pdf |
| 2020-10-16,Fri | Prohibition of crossing statistical regions with most infected (red list) | https://www.uradni-list.si/\_pdf/2020/Ur/u2020143.pdf |
| 2020-10-17,Sat | Contact tracing stopped | https://www.nijz.si/spremenjen-protokol-sledenja-stikom-okuzenih-z-novim-koronavirusom-sars-cov-2 |
| 2020-10-19,Mon | School (from 6th grade) and music school closures | https://www.gov.si/novice/2020-10-15-ministri-hojs-kustec-in-pocivalsek-ter-drzavna-sekretarka-bregant-predstavili-ukrepe-glede-na-epidemiolosko-sliko-v-rdecih-regijah/ |
| 2020-10-19,Mon | Proclamation of epidemics | https://www.uradni-list.si/\_pdf/2020/Ur/u2020146.pdf |
| 2020-10-20,Tue | Police hour (9 pm - 6 am), prohibition of crossing among statistical regions, prohibition of gatherings of more than 6 people, prohibition of all events | https://www.uradni-list.si/\_pdf/2020/Ur/u2020147.pdf |
| 2020-10-24,Sat | Closure of bars, hotels, restaurants, shopping malls, hairdressers, beauty salons; restricted public transport | https://www.gov.si/novice/2020-10-22-predsednik-vlade-janez-jansa-pred-nami-je-teden-boja-za-zaustavitev-sirjenja-virusa-med-nami/ |
| 2020-10-26,Mon | Testing strategy: only vulnerable groups and those with anticiapted severe disease course | https://zd-sentjur.si/wp-content/uploads/2020/10/Nova\_navodila\_glede\_testiranja\_in\_i\_P.pdf |
| 2020-10-26,Mon | Schools and child-care closed | https://www.gov.si/novice/2020-10-22-predsednik-vlade-janez-jansa-pred-nami-je-teden-boja-za-zaustavitev-sirjenja-virusa-med-nami/ |
| 2020-10-27,Tue | Prohibition of crossing municipality-of-residence borders | https://www.uradni-list.si/\_pdf/2020/Ur/u2020155.pdf |
| 2020-11-06,Fri | Testing strategy: all | https://www.gov.si/novice/2020-11-06-spremenjen-nacin-testiranja-na-covid-19/ |
| 2020-11-13,Fri | Prohibition of all gatherings (but household members) | https://www.uradni-list.si/\_pdf/2020/Ur/u2020163.pdf |
| 2020-11-16,Mon | Prohibition of goods selling and services to consumers (except vital: groceries, pharmacies, post, banks) | https://www.uradni-list.si/\_pdf/2020/Ur/u2020163.pdf |
| 2020-11-16,Mon | Prohibition of public transport | https://twitter.com/JernejVrtovec/status/1326942175519760384 |
| 2020-12-07,Mon | Prohibition of alcohol serving/acceptance at take-away points of bars/restaurants | https://www.gov.si/novice/2020-12-04-pojasnilo-glede-osebnega-prevzema-alkohola-in-alkoholnih-pijac/ |
