# Supplementary material for: COVID-19 in Slovenia, from a Success Story to Disaster: What Lessons Can Be Learned?
Source: Life (Basel). 2021 Oct 4;11(10):1045. doi: 10.3390/life11101045 (PMC8541564; doi:10.3390/life11101045)
Supplement: Supplementary file 1 [file life-11-01045-s001.zip › Table_S2.pdf]

Table S2: Estimated number of cumulative deaths per 1 million inhabitants per 14-days (MF) and the largest number of patients treated in hospitals and ICUs during the second wave up to December 1, 2020 under counterfactual scenarios (1)-(7), see the paper for detailed description.

Scenario		Deaths (cumulative)		Hospitals (day max)		ICU (day max)	
Mobility	C-T	Est.	90% CI	Est.	90% CI	Est.	90% CI
(1) real	(a)	54	[50-57]	1338	[1256-1430]	223	[212-234]
	(b)	14	[12-16]	281	[245-322]	48	[41-55]
(2) pre-COVID	(a)	439	[230-684]	9654	[3366-17871]	1647	[561-3066]
	(b)	278	[147-452]	5260	[2562-8664]	888	[434-1461]
(3) stopped	(a)	0.1	[0.1-0.2]	6	[4-7]	1	[1-1]
	(b)	0.1	[0.1-0.2]	6	[4-7]	1	[1-1]
(4) after LD	(a)	37	[34-41]	983	[940-1029]	170	[162-177]
as first wave	(b)	13	[11-15]	281	[245-322]	48	[41-55]
(5) LD (expected)	(a)	11	[7-16]	169	[113-271]	29	[19-46]
on Sept 14	(b)	9	[6-13]	169	[113-271]	29	[19-46]
(5.1) LD (instant)	(a)	5	[4-7]	66	[62-70]	11	[11-12]
on Sept 14	(b)	4	[3-4]	66	[62-70]	11	[11-12]
(6) LD (expected)	(a)	2	[1-2]	35	[28-46]	6	[5-8]
on Aug 10	(b)	2	[1-2]	35	[28-46]	6	[5-8]
(6.1) LD (instant)	(a)	1	[1-1]	20	[19-21]	3	[3-4]
on Aug 10	(b)	1	[1-1]	20	[19-21]	3	[3-4]
(7) decreased	(a)	7	[7-9]	160	[151-170]	27	[26-29]
on Oct 1	(b)	7	[6-7]	160	[152-169]	27	[26-29]

(a) C-T stops on October 9 (as in reality); (b) C-T is performed throughout the period
C-T = contact tracing, LD = lockdown, Est. = estimate (posterior mode), CI = credible interval
