# Supplementary figures and images for: COVID-19 in Slovenia, from a Success Story to Disaster: What Lessons Can Be Learned?
Source: Life (Basel). 2021 Oct 4;11(10):1045. doi: 10.3390/life11101045 (PMC8541564; doi:10.3390/life11101045)

First wave

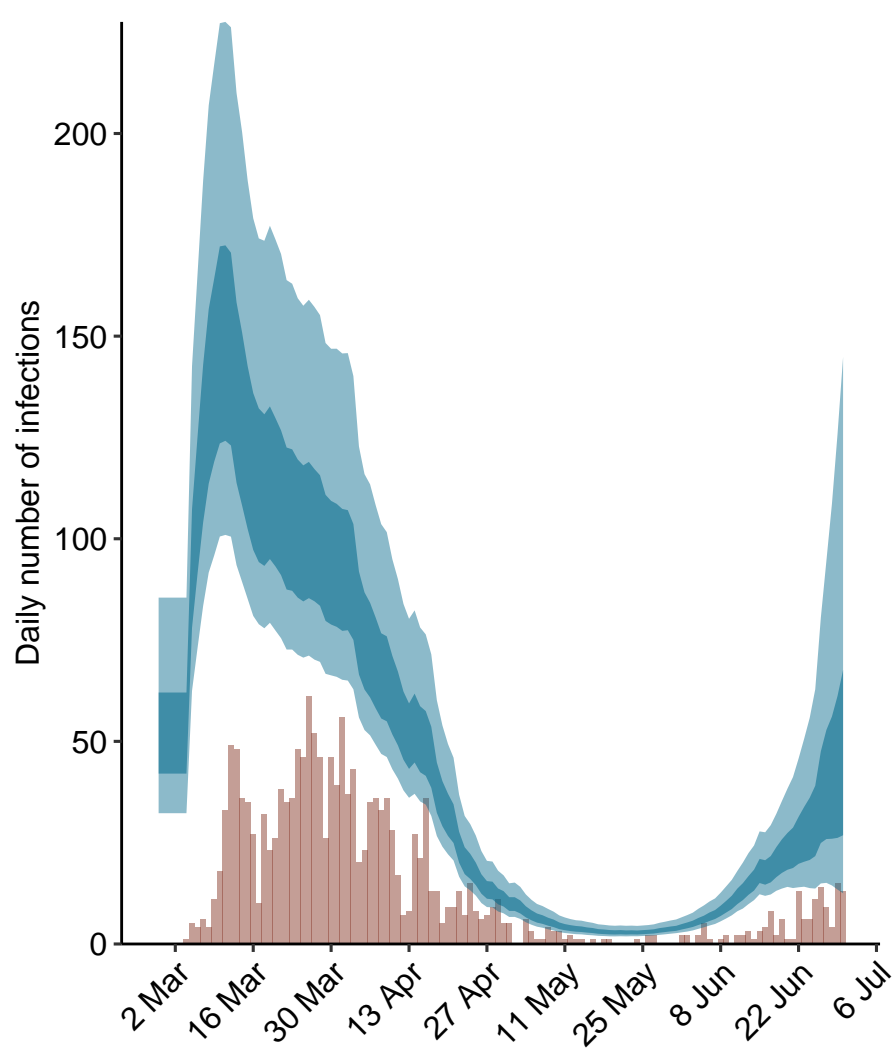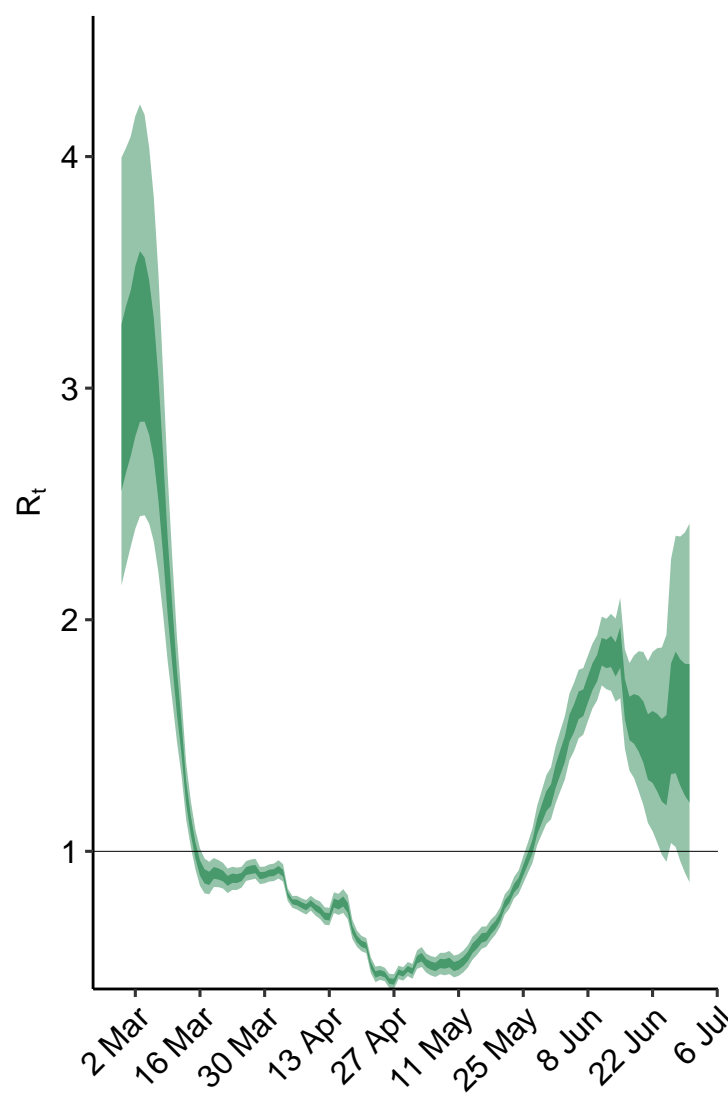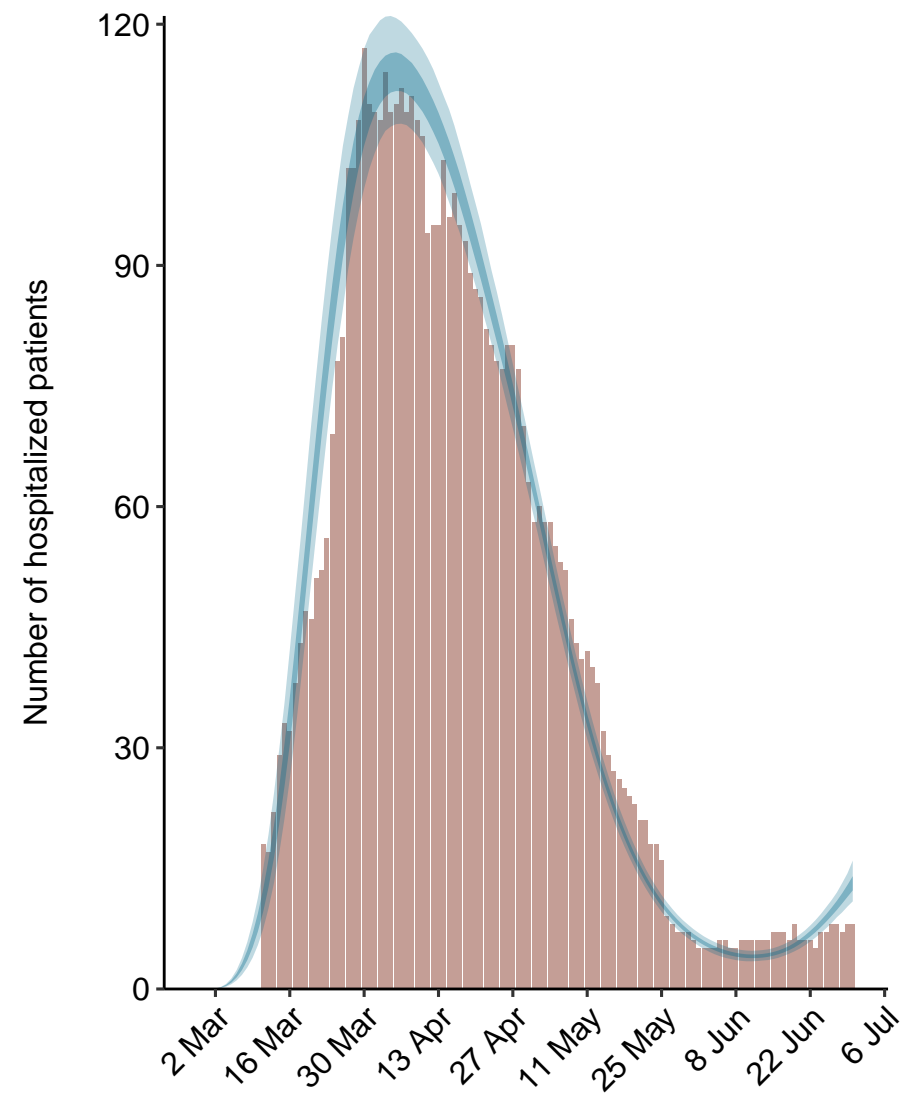

Second wave

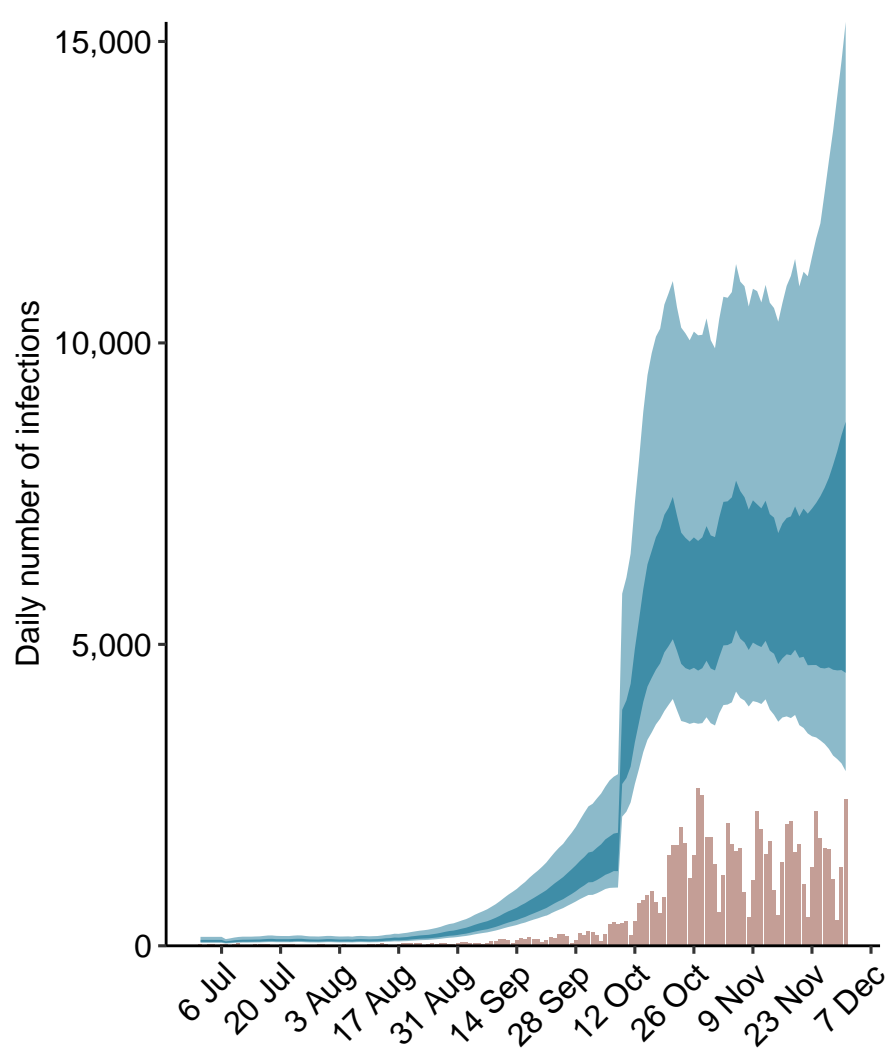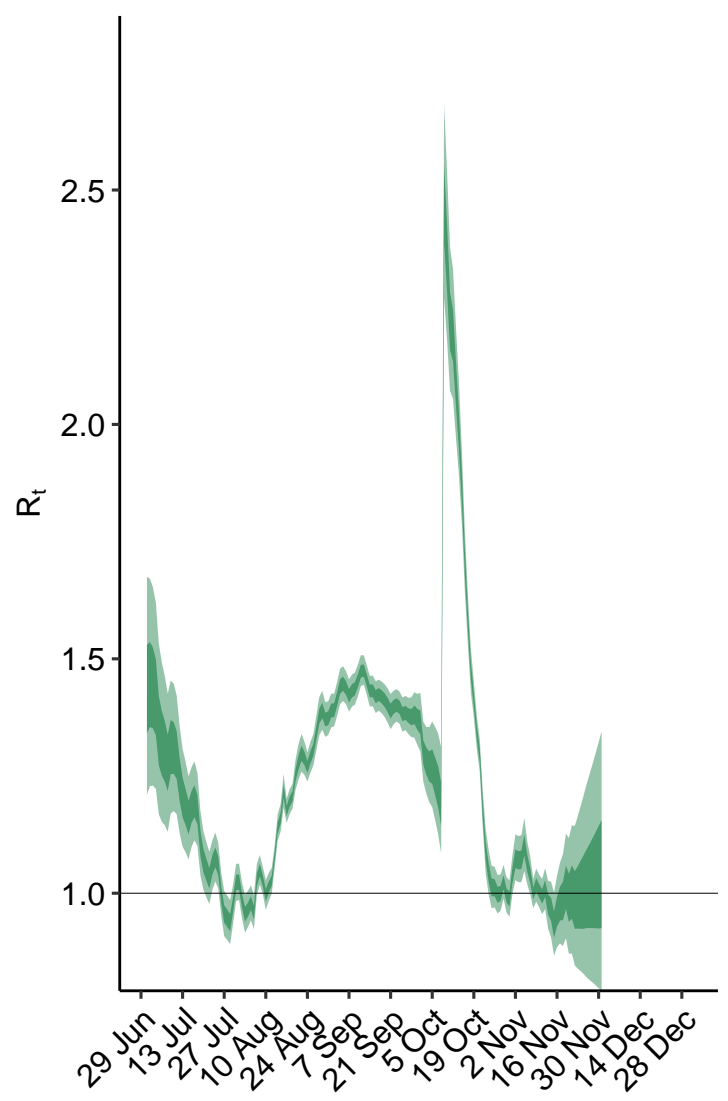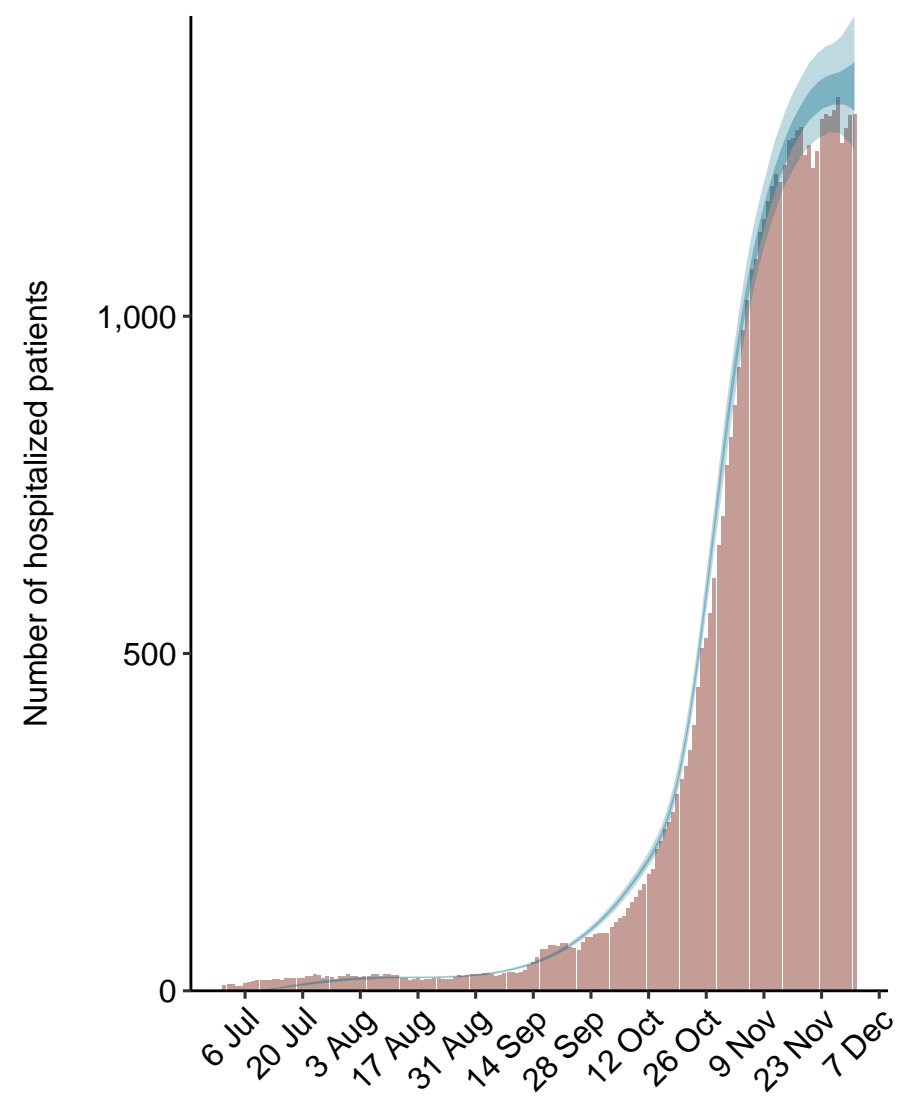

Supplement: Supplementary file 1 [file life-11-01045-s001.zip › Figure_S1.pdf]
